# Barriers and facilitators of artificial intelligence conception and implementation for breast imaging diagnosis in clinical practice: a scoping review

**DOI:** 10.1007/s00330-023-10181-6

**Published:** 2023-09-02

**Authors:** Belinda Lokaj, Marie-Thérèse Pugliese, Karen Kinkel, Christian Lovis, Jérôme Schmid

**Affiliations:** 1 Geneva School of Health Sciences, HES-SO University of Applied Sciences and Arts Western Switzerland, Delémont, Switzerland; 2 Faculty of Medicine, University of Geneva, Geneva, Switzerland; 3 Division of Medical Information Sciences, Geneva University Hospitals, Geneva, Switzerland; 4 Réseau Hospitalier Neuchâtelois, Neuchâtel, Switzerland

**Keywords:** Breast neoplasms, Diagnostic imaging, Artificial intelligence, Deep learning

## Abstract

**Objective:**

Although artificial intelligence (AI) has demonstrated promise in enhancing breast cancer diagnosis, the implementation of AI algorithms in clinical practice encounters various barriers. This scoping review aims to identify these barriers and facilitators to highlight key considerations for developing and implementing AI solutions in breast cancer imaging.

**Method:**

A literature search was conducted from 2012 to 2022 in six databases (PubMed, Web of Science, CINHAL, Embase, IEEE, and ArXiv). The articles were included if some barriers and/or facilitators in the conception or implementation of AI in breast clinical imaging were described. We excluded research only focusing on performance, or with data not acquired in a clinical radiology setup and not involving real patients.

**Results:**

A total of 107 articles were included. We identified six major barriers related to data (B1), black box and trust (B2), algorithms and conception (B3), evaluation and validation (B4), legal, ethical, and economic issues (B5), and education (B6), and five major facilitators covering data (F1), clinical impact (F2), algorithms and conception (F3), evaluation and validation (F4), and education (F5).

**Conclusion:**

This scoping review highlighted the need to carefully design, deploy, and evaluate AI solutions in clinical practice, involving all stakeholders to yield improvement in healthcare.

**Clinical relevance statement:**

The identification of barriers and facilitators with suggested solutions can guide and inform future research, and stakeholders to improve the design and implementation of AI for breast cancer detection in clinical practice.

**Key Points:**

• *Six major identified barriers were related to data; black-box and trust; algorithms and conception; evaluation and validation; legal, ethical, and economic issues; and education*.

• *Five major identified facilitators were related to data, clinical impact, algorithms and conception, evaluation and validation, and education*.

• *Coordinated implication of all stakeholders is required to improve breast cancer diagnosis with AI*.

**Supplementary Information:**

The online version contains supplementary material available at 10.1007/s00330-023-10181-6.

## Introduction

Although AI has demonstrated promise in enhancing breast cancer diagnosis, the implementation of AI algorithms in clinical practice encounters various barriers. This scoping review aims to identify these barriers and facilitators to highlight key considerations for developing and implementing AI solutions in breast cancer imaging. The first modality that incorporated AI techniques through traditional computer-aided detection (CAD) was mammography (MG) [1]. CAD was initially developed to assist radiologists in the detection of breast cancers they would have potentially missed without the help of CAD. However, a large study including more than 495,000 digital screening mammograms compared the performance of screening mammography with and without CAD by 271 radiologists, and it was demonstrated that screening performance was not improved with traditional CAD systems [2], and therefore there was no clinical benefit for patients. Over the last decade, advances in AI have encouraged the clinical study and implementation of AI-based CAD because it offers superior detection performance while not being reliant on hand-crafted imaging features [3]. Many studies demonstrated the good performance of AI in the detection of breast cancer using MG, ultrasound (US), or magnetic resonance imaging (MRI) — with similar performance to radiologists [4–8]. Furthermore, when AI is used by radiologists, there is less variability in radiologists’ interpretations, regardless of their experience, leading to a more reproducible and standardized diagnosis [4, 7]. Finally, AI is capable of processing and analyzing images faster than radiologists. This advantage is particularly pronounced when considering MRI, a multi-parametric modality, that comprises a lot of sequences, which considerably reduces reading time. In the case of screening programs with MG resulting in a large number of examinations, AI can be used for mammogram triage by automatically detecting normal exams, allowing radiologists to focus on other more complex exams [9]. The great need for data quality and quantity constitutes a disadvantage because if there is not enough data or using insufficient quality, AI models can be biased. The quantity of available data differs among modalities; digital breast tomosynthesis (DBT) and MG are typically favored because of their large accessibility [10]. Another disadvantage concerns the external validation of a model; most models are trained and developed from a single dataset and therefore cannot be applied easily to different populations or clinical settings [11]. AI can offer both advantages and disadvantages to breast imaging, and more precisely the barriers and facilitators of the implementation of such systems in clinical breast imaging settings have not been clearly stated. While previous scoping reviews have partially addressed barriers to implementing AI in breast cancer imaging [12, 13], they have not provided a comprehensive overview of these barriers. This study aims to fill this gap by identifying and categorizing the key barriers and facilitators to developing and implementing AI solutions for cancer detection in clinical breast imaging practice.

## Method

Literature research was conducted in six databases (PubMed, Web of Science, CINHAL, Embase, IEEE, ArXiv) to get exhaustive results. The search was limited to 10 years (2012–2022) to target the recent advancement of AI in clinical breast imaging. Published articles of any design were eligible. We excluded conference abstracts, as they do not contain sufficient data for this review. Search strategy details were designed by M.P. and can be found in Supplement S1.

**Fig. 1:**
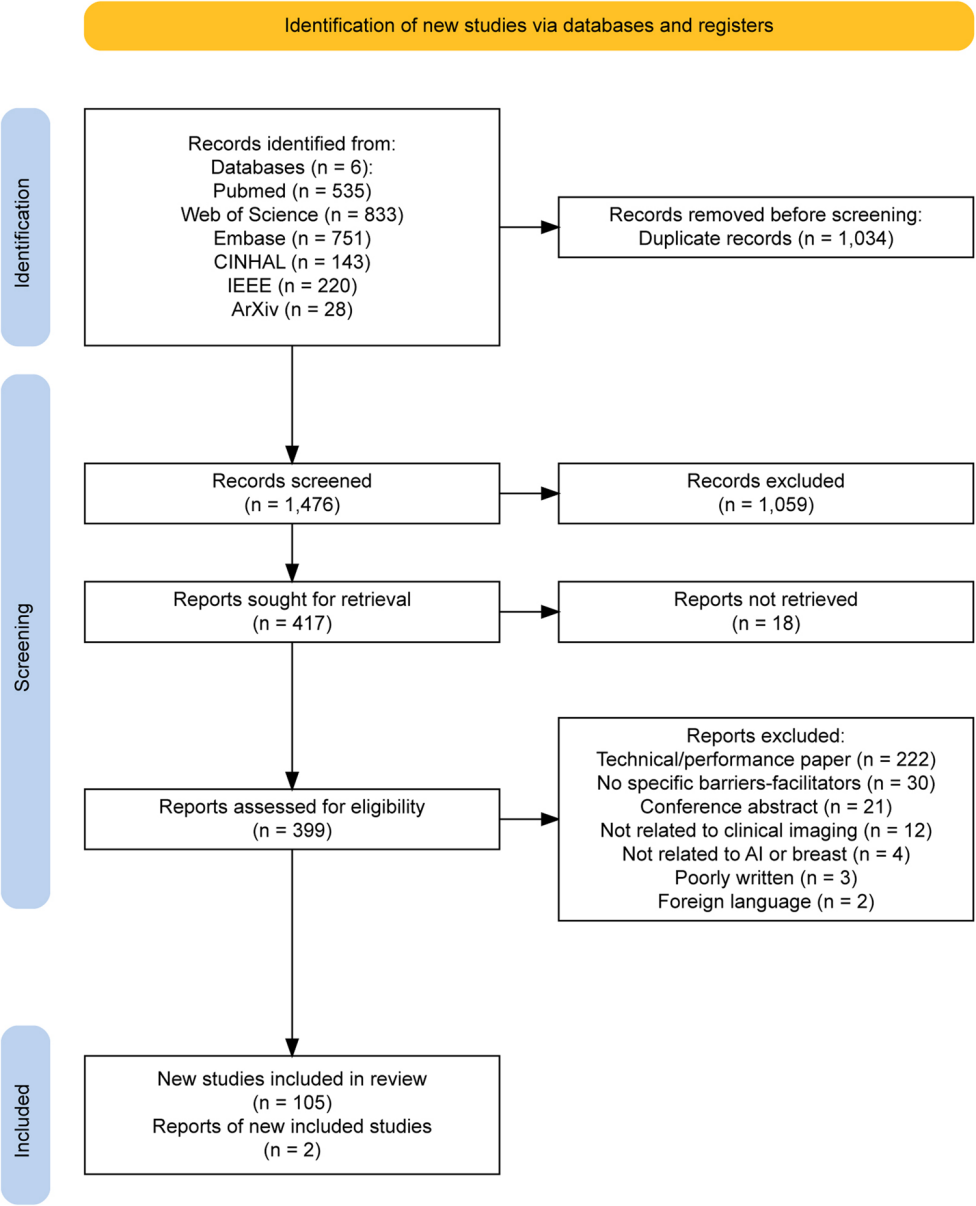
PRISMA flow diagram

The selection of studies was done in two stages as depicted in the PRISMA [14] flow (Fig. 1), with the RAYYAN tool [15]. For the first stage, two reviewers (J.S., B.L.) independently screened the title and abstract of each article. Then in the second stage, the selected studies, as well as additional works found through screening the reference lists, were independently reviewed based on the inclusion criteria. When there were disagreements between the two reviewers, divergences were discussed and solved. There was no need for a third reviewer. Papers were included when there was mention of barriers and/or facilitators of AI for cancer detection in breast imaging. The following criteria led to the exclusion of papers:

- Focus only on performance,
- Use of synthetic datasets or data acquired with phantoms,
- Not conventional clinical imaging of the breast (e.g., thermal imaging, microwave breast imaging),
- Focus on histopathology images,
- Conference abstract.

Papers that could not be found or were not available were also excluded. The final included studies were charted in a table containing structured information and characteristics of the studies (e.g., author, year of publication, geographical information, paper type, modality) (Supplement Table S4).

## Results

### Characteristics of the included publications

**Fig. 2:**
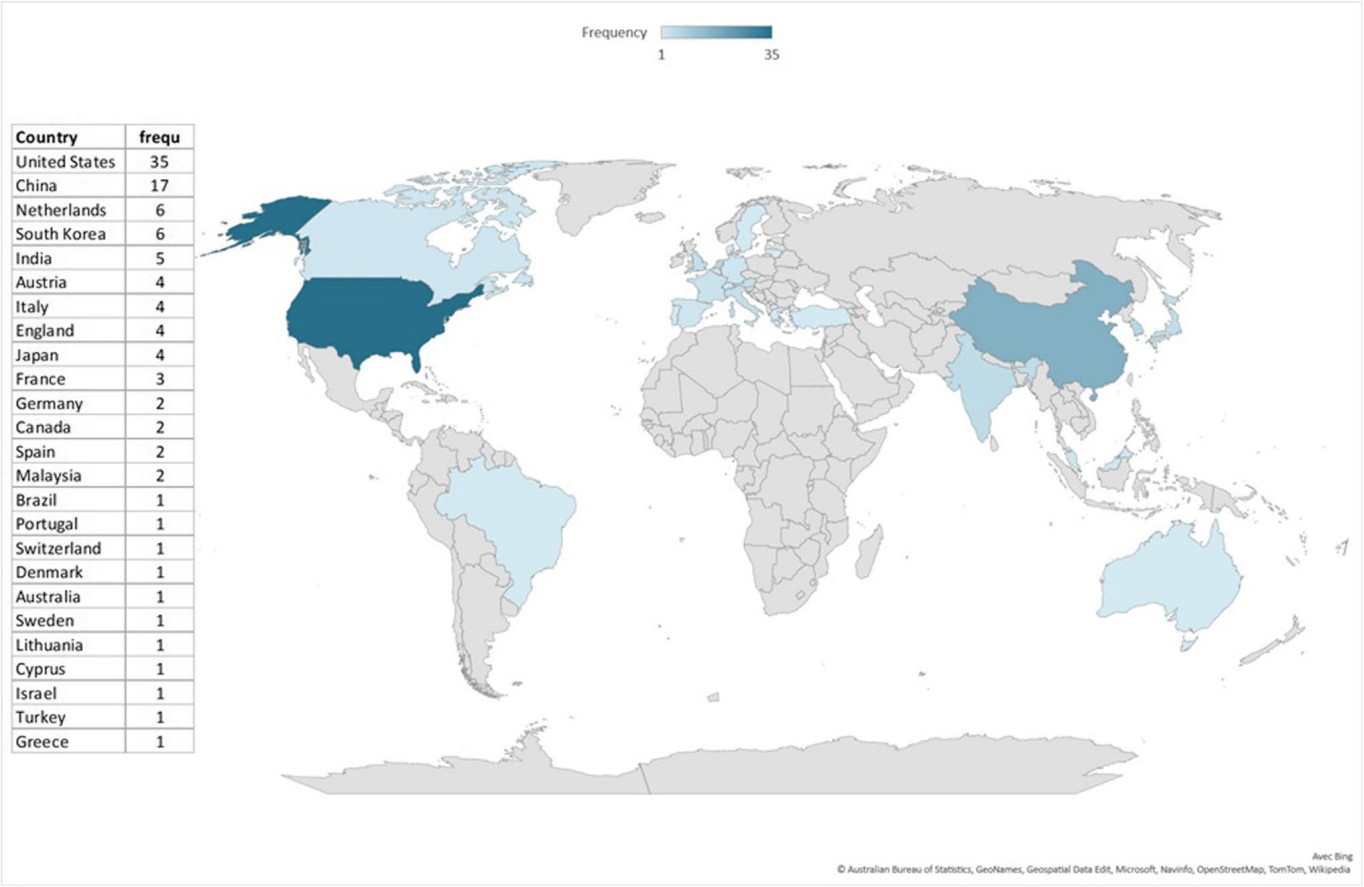
Geographic distribution of included articles

**Fig. 3:**
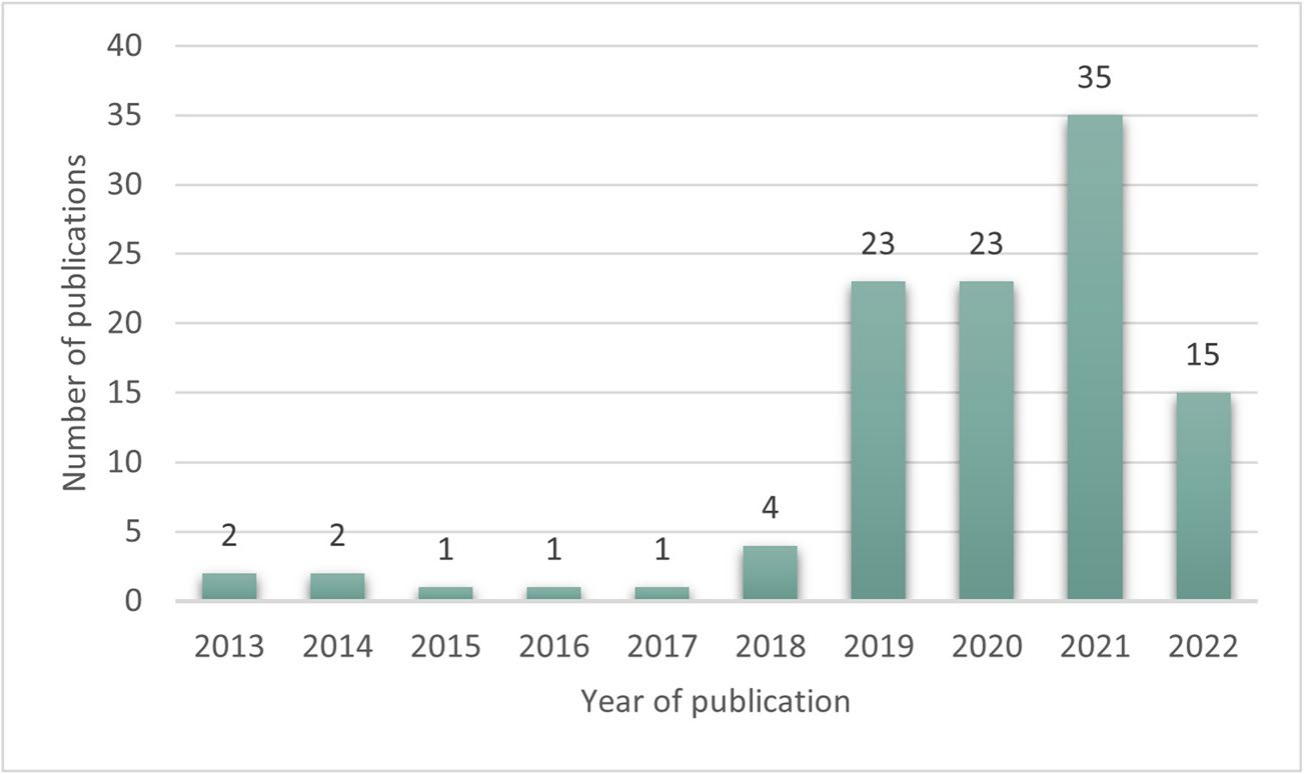
Temporal distribution of included articles from 2012 to 2022

**Fig. 4:**
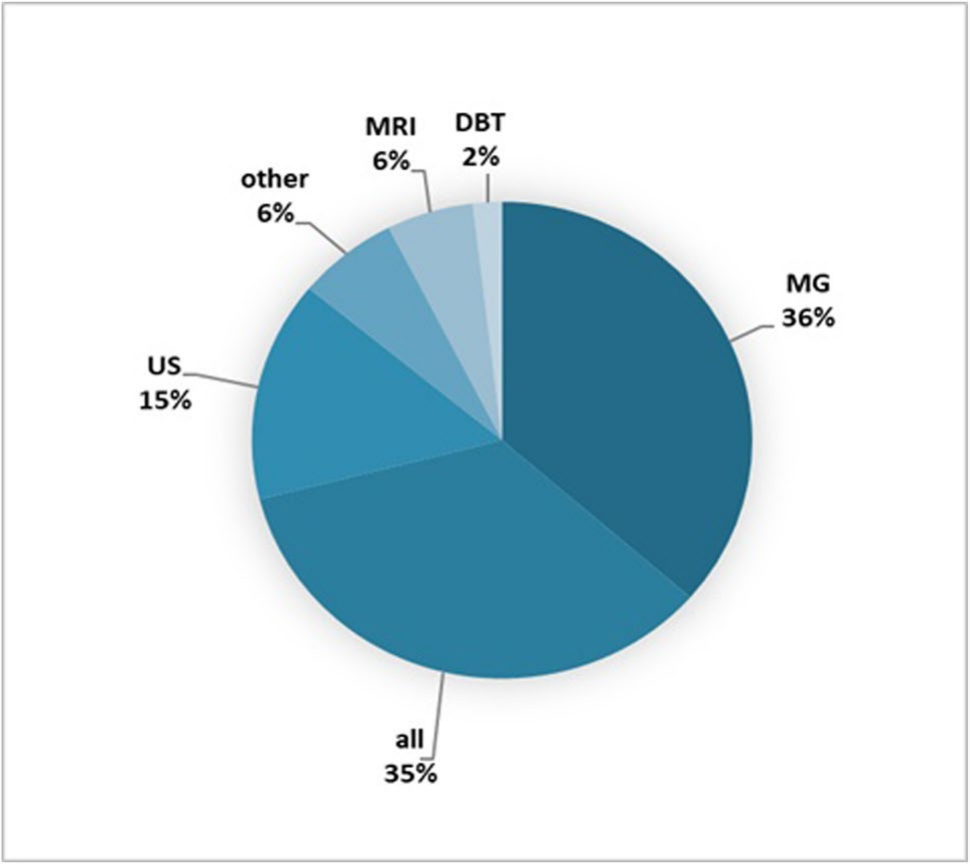
Method distribution among publications (MG, digital mammography (36%); US, ultrasound (15%); MRI, magnetic resonance imaging (6%); DBT, digital breast tomosynthesis (2%); all, breast imaging techniques in general (35%); other, two or three breast imaging modalities (6%))

**Fig. 5:**
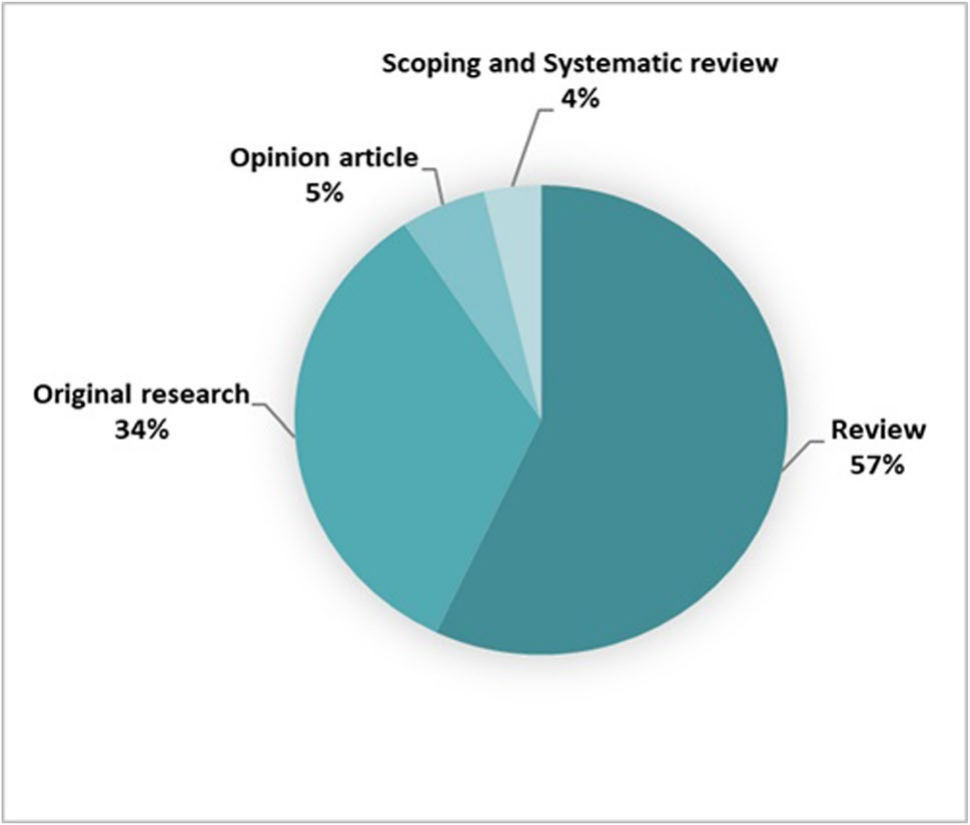
Type of publications (opinion article (5%) includes perspective articles, editorial comments, and case study)

Of the 1476 screened publications, 107 were included (Fig. 1). Most papers originated from the USA (*n* = 37) and China (*n* = 17) (Fig. 2), and a large proportion (89.7%) were published after 2018 (Fig. 3). As depicted in Fig. 4, most articles focused on a single modality including MG (*n* = 39), US (*n* = 16), breast MRI (*n* = 6), and DBT (*n* = 2). In the “other” category, the studies were classified involving two or three modalities (*n* = 7) such as CT and US (*n* = 1), MG and DBT (*n* = 4), MRI and US (*n* = 1), and CT and MRI and US (*n* = 1). The “all” category referred to the inclusion of breast imaging as a whole, mentioning all the modalities in general (*n* = 37). Figure 5 shows that most of the articles were review articles (57%), and original research papers (34%). Scoping (*n* = 2) and systematic reviews (*n* = 2) (4%), as well as some opinion articles (*n* = 6, 5%), were included. Very few articles made their data (*n* = 6) or code (*n* = 8) publicly available or available under request to the authors. Details are provided in Supplement Table S4.

### Main identified barriers and facilitators of AI in breast clinical imaging

**Fig. 6:**
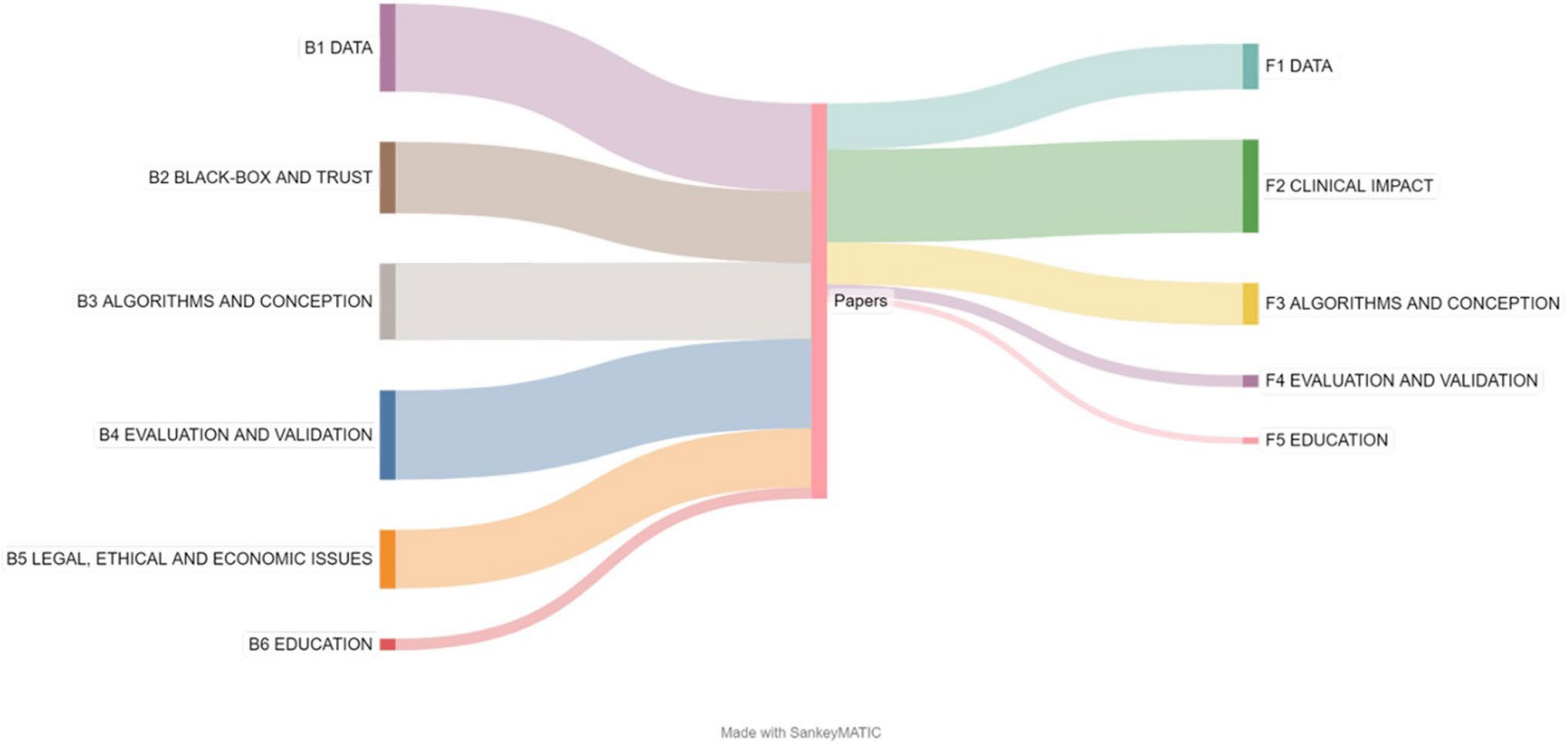
Sankey diagram representation of major barriers (left) and facilitators (right) distribution among included papers

Barriers and facilitators were identified through the first screening stage, and then clarified and enriched iteratively through complete text analysis of the included studies. Detailed tables can be found in Supplement Tables S2 and S3. Table 1 reports the main identified barriers and facilitators derived from included papers with relative frequency. We identified six major barriers involving data (B1), black box and trust (B2), algorithms and conception (B3), evaluation and validation (B4), legal, ethical, and economic issues (B5), and education (B6). We determined five major facilitators involving data (F1), clinical impact (F2), algorithms and conception (F3), evaluation and validation (F4), and education (F5). Figure 6 highlights that major barriers were reported twice as much as major facilitators. Sub-barriers and sub-facilitators were also derived as described in the following.

**Table 1:** Identified barriers and facilitators

Barriers		Number of paper (%)	Facilitators		Number of paper (%)
**B1**	**DATA**		**F1**	**DATA**	
B1.1	Data size and variety	60 (56.1%)	F1.1	Datasets initiatives	15 (14%)
B1.2	Data quality and data processing	48 (44.9%)	F1.2	Algorithmic approaches to address data barriers	27 (25.2%)
B1.3	Data sharing	15 (14.0%)	**F2**	**CLINICAL IMPACT**	
**B2**	**BLACK-BOX AND TRUST**		F2.1	Diagnostic performance	53 (49.5%)
B2.1	Model transparency	51 (47.7%)	F2.2	Clinical workflow	58 (54.2%)
B2.2	Clinician trust	19 (17.8%)	**F3**	**ALGORITHMS AND CONCEPTION**	
B2.3	Patient trust	11 (10.3%)	F3.1	Multivariable data	24 (22.4%)
**B3**	**ALGORITHMS AND CONCEPTION**		F3.2	Numerous algorithms	11 (10.3%)
B3.1	Model architecture	16 (15.0%)	**F4**	**EVALUATION AND VALIDATION**	
B3.2	Technical constraints	17 (15.9%)	F4.1	Increased accessibility of AI	3 (2.8%)
B3.3	Multivariable data	28 (26.2%)	F4.2	Benchmarking of AI approaches	6 (5.6%)
B3.4	Involvement of stakeholders	29 (27.1%)	**F5**	**EDUCATION**	
**B4**	**EVALUATION AND VALIDATION**		F5.1	AI for education	5 (4.6%)
B4.1	Meaningful clinical evaluation	44 (41.1%)			
B4.2	Data variability	54 (50.5%)			
B4.3	Quality assurance	17 (15.9%)			
**B5**	**LEGAL, ETHICAL AND ECONOMIC ISSUES**				
B5.1	Liability	22 (20.6%)			
B5.2	Law and policies	24 (22.4%)			
B5.3	Fair AI	22 (20.6%)			
B5.4	Cybersecurity	10 (9.3%)			
B5.5	Economic issues	11 (10.3%)			
**B6**	**EDUCATION**				
B6.1	User education	9 (8.4%)

#### B1 Data

##### Data size and variety (B1.1)

The majority of publications (*n* = 60) reported the problem of small datasets for training and validation, as the size of the datasets was relatively small [16] to properly train AI algorithms. Different mentioned causes were limited data availability due to privacy concerns or technical constraints (e.g., collection of paired imaging data) [17, 18], insufficient data diversity (variety of population and imaging protocols, pathology nuances, etc.) often leading to overfitting, or data balancing issues (under/over-represented categories in the dataset, commonly observed in the medical field and that can affect prediction accuracy) [11, 16, 17, 19–22].

##### Data quality and data processing (B1.2)

Linked to data quantity, model performance depends on data quality affected by noisy or missing/incomplete data. This barrier was often mentioned (*n* = 48) as it requires appropriate data labelling supported by reliable annotation methods, which can be a very demanding process (need for trained staff to label or verify the data). This process was qualified as expensive, time-consuming, and subjective if not adequately performed [18, 19, 23–28].

##### Data sharing (B1.3)

The ability to share and pool data across actors (institutions, research teams, etc.) was reported as limited (*n* = 15). These limitations concern patient confidentiality, regulations, institutional policies, or different interests of stakeholders [10, 23, 29, 30].

#### B2 Black box and trust

##### Model transparency (B2.1)

The barrier of model transparency was often mentioned (*n* = 51). There is a need to develop transparent models able to explain what is predicted, how it has been done, and the degree of prediction confidence. Thus, it is important to have an interpretable AI system (the rationale followed by the system) to detect possible biases generated by the algorithms [11, 31, 32]. In the literature, the terms “transparency”, “explainability”, and “interpretability” are often used interchangeably, and there are no generally accepted definitions; therefore, all three terms were regrouped.

##### Clinician trust (B2.2)

Related to the interpretability problem, the adoption of an AI system depends on the level of confidence that the radiologists have in the AI system and its findings, and also on the understanding they have about the tool (*n* = 19) [24, 33, 34]. Avoiding dependence on AI systems to preserve users’ ability to think critically and make good patient decisions is also linked to radiologists’ trust [35, 36].

##### Patient trust (B2.3)

Patient confidence (*n* = 11) in the AI systems was also reported as important in the same way as for radiologists, and is also linked to communication between human and machine when the diagnosis is disclosed [3, 37].

#### B3 Algorithm and conception

##### Model architecture (B3.1)

A large variety of AI algorithmic models exists, along with multiple ways for parameterizing, training, validating, and testing these models. This contributes to the difficulty of choosing and tuning the right architecture for a given purpose, as well as devising objective ways to compare results between models (*n* = 16) [20, 26, 38].

##### Technical constraints (B3.2)

Model architectures may impose some constraints (*n* = 17) on the input data (data dimensionality, resolution, layout, etc.) leading to data transformation (e.g., data dimensionality reduction) [10]. Similarly, hardware constraints (storage, memory, computing capabilities, etc.) may limit the choice of usable models or further contribute to data transformation [26, 38–41]. Finally, difficulties related to the integration of AI tools in clinical practice (e.g., compatibility with PACS and RIS systems, characteristics of the imaging modalities) are part of these technical constraints [33, 42, 43].

##### Multi-variable data (B3.3)

The development of efficient algorithms is more challenging due to the complex nature of multi-variable data essential for clinical reasoning. This data includes multi-modality imaging, multi-parametric protocols, clinical knowledge, and previous or contralateral examinations (*n* = 28) [29, 44].

##### Involvement of stakeholders (B3.4)

There is a need for a closer collaboration (*n* = 29) between all stakeholders (clinicians, data scientists, researchers, industry, policymakers, patients, etc.) in the design and development of AI systems [1, 19, 33, 45], sharing of development tools and procedures, as well as in the definition of common definitions and metrics for evaluation and model comparison [18, 27, 28].

#### B4 Evaluation and validation

##### Meaningful clinical validation (B4.1)

Assessments of clinical utility, performance, and adoption are of paramount importance and were thus often mentioned in the studies (*n* = 44). Prospective studies are required to evaluate performance and the effect on clinicians in clinical settings, distinguishing engineering metrics from clinical ones [11], as well as the type of clinical scenarios (e.g., screening vs. follow-ups). Large trials should be conducted, possibly over long periods, and the use of independent test datasets was promoted [1, 3, 19, 28].

##### Data variability (B4.2)

In real-world clinical practice, there is inherent data variability depending on e.g. manufacturers, equipment age and characteristics, imaging protocols, and population traits that must be taken into account to assess the generalization of the deployed AI systems (*n* = 54) [10, 19, 27].

##### Quality assurance (QA) (B4.3)

Continuous monitoring of AI algorithms was recommended (*n* = 17) for continuous improvements or to prevent performance degradation over time due to changes, requiring to put in place QA procedures with adequate resources [40, 43, 46].

#### B5 Legal, ethical, and economic issues

##### Liability (B5.1)

Many open questions are raised concerning the final responsibility in the decisions made for patient care (*n* = 22) (e.g., Can final decisions be made by the AI systems? Who will be responsible for errors? Will AI negatively influence radiologists?), and thus these questions need to be addressed [11, 42].

##### Law and policies (B5.2)

This barrier (*n* = 24) highlighted concerns about the importance of patient-privacy policies, market approval or clearance of AI solutions, intellectual properties (e.g., who owns the data?), and regulatory guidelines [11, 19, 33].

##### Fair AI (B5.3)

There are many ways to unintentionally introduce biases in AI systems (e.g., in data collection or development stages) and it is crucial to embrace fair AI practices promoting equity in diagnosis and treatment across diverse populations without excluding minorities (*n* = 22) [21, 28, 30].

##### Cybersecurity (B5.4)

Few studies reported concerns about cybersecurity (*n* = 10). Healthcare data being very sensitive, the importance of addressing security risks, preventing imaging data sabotage/manipulation (e.g., adversarial attacks by inserting or removing pathologies), and ensuring data protection and privacy were reported [3, 19].

##### Economic issues (B5.5)

Economic barriers were mentioned in some papers (*n* = 11), due to different interests among stakeholders (e.g., company vs. researchers), reimbursement policies (e.g., CAD is reimbursed in the USA but not in EU for most cases, with the potential exception of screening) [47], increased health costs due to unnecessary actions (non-relevant findings leading to overdiagnosis or overtreatment) [48], costs of collecting and processing data, especially from expensive/less common modalities such as MRI, and the costs of running and maintaining AI systems [23, 33, 45].

#### B6 Education

##### User education (B6.1)

Few papers (*n* = 5) reported that users (radiologists, patients, health professionals, etc.) must have sufficient knowledge about AI data (collection, annotation, etc.) and AI tools (terminologies and concepts, methods, and applications, etc.) to critically evaluate them, and be aware of their strengths and limitations [9, 11, 33, 35].

#### F1 Data

##### Datasets initiatives (F1.1)

There exist initiatives from multiple public and private sector institutions to create large, diversified, annotated datasets, to be made available to public databases, challenges, etc. (*n* = 15) [19, 23, 32, 39, 49].

##### Algorithmic approaches to address data barriers (F1.2)

Some solutions were mentioned or utilized to try to address the issues of lack of or non-shareable data (*n* = 27) [28], such as federated learning (data remains locally but algorithm parameters travel [10, 19]), swarm learning [10], transfer learning [16, 26], data augmentation [49], data normalization [10], and generative models [39].

#### F2 Clinical impact

##### Diagnostic performance (F2.1)

A model/AI system’s performance is evaluated using a test set. In the different studies, the metrics usually used are area under the ROC curve (AUC), or sensitivity and specificity, with or without comparison to the radiologist or in combination with the radiologist. Generally, good diagnostic performance was reported in surveyed reviews and original research works (*n* = 53) [5, 8, 50, 51].

##### Clinical workflow (F2.2)

A lot of papers (*n* = 58) reported that AI systems positively impacted clinical workflow by improving efficiency in clinical practice, such as triaging, using AI as proof reader, reading time reduction, improved communication with patients, reduction of radiologist fatigue, cost reduction, or more reproducible readings [9, 11, 12, 52, 53].

#### F3 Algorithms and conception

##### Multi-variable data (F3.1)

Some papers (*n* = 24) argued that multi-variable data (e.g., multi-modality imaging, multi-parametric protocols, or inclusion of clinical non-imaging information) could lead to better results for AI tools because each source of data provides valuable information and machine learning (ML) offers approaches to embrace multi-dimensional data [10, 13, 37, 54].

##### Numerous algorithms (F3.2)

It was also reported that the large number of algorithms developed provides greater flexibility for AI integration (*n* = 11). The use of ensemble models combining the predictions of several algorithms has also been reported as a way to increase performance [21, 32, 55, 56].

#### F4 Evaluation and validation

##### Increased accessibility of AI (F4.1)

There is an increasing number of (certified) AI products that are made available in clinical practice, facilitating the setup of clinical trials and prospective studies (*n* = 3) [19, 34, 37].

##### Benchmarking of AI approaches (F4.2)

There were mentioned (*n* = 6) international scientific challenges that contribute to strengthening the benchmarking of AI approaches by providing open and reproducible evaluation (training and testing data, evaluation metrics, and tools) that strongly encourages or imposes the sharing of computer code from participants [47, 57].

#### F5 Education

##### AI for education (F5.1)

Few papers (*n* = 5) highlighted that some systems or models can be used for the education of clinicians, such as generative models producing images for the training of radiologists [39, 50] or systems giving feedback to radiographers for image quality or acquisition parameters [48].

## Discussion

This scoping review provides a comprehensive summary of the barriers and facilitators encountered during the creation and deployment of AI in clinical settings. Solutions can then be found and measures can be taken for better implementation of AI systems in practice — bringing tangible benefits to both patients and clinicians.

### Conception

The importance of data size (B1.1) and data quality (B1.2) was predominant in the included studies, being directly related to the effectiveness of machine learning and the issue of data sharing (e.g., less than 17% of original papers’ data is publicly available or under request as shown in Supplement Table S4). As a result of screening programs and higher availability, it was highlighted that digital MG and DBT studies usually have large datasets (many thousands of patients, from 9919 to 32,714 women in studies with MG [58] and DBT [51]) often acquired in several centers, in comparison to studies involving US and MRI [1, 10]. MRI studies especially lacked data (from 93 [59] to 1715 patients [25] in included studies, with varying types of MR protocols). While US imaging may benefit from larger accessibility, an important discrepancy was observed among studies where the number of patients ranged from 92 to 5151 [54, 60–67]. Variety in the data was also reported as a critical issue since clinical data is often imbalanced due to disease prevalence, data availability, or population characteristics, which remains an unsolved issue still under research in the ML field [16]. In practice, data curation is a difficult and tedious task, especially depending on the type of collected data or modality of imaging. Collection without interfering with the clinical practice workflow, anonymization process, ethics commission requirements, and compliant and robust databases are among the many hurdles faced during this process. Despite being expensive and time-consuming, data curation with clinical expert validation remains an absolute necessity [68].

To address these challenges, some solutions can be considered, like large datasets initiatives (F1.1) (e.g., result of competitions opened to the research community [57, 69]), more adapted algorithmic approaches (F1.2), or the promotion of data sharing and collaboration between clinical settings by supporting regulations on patient confidentiality in favor to research (such as the generalized patient consent). These actions can contribute to having larger but also more representative and diversified datasets.

To support data annotation, one suggestion would be to rely more on automated systems compliant with regulations and ease the work of clinical annotators. For instance, pathological findings reported in radiological reports could be associated with their exact localization in the images via user-friendly reporting tools, simplifying the data annotation and expediting its processing. In the context of creating a data challenge on US breast lesions, Lassau et al [57] observed the added value of relying on professional tools to anonymize and gather annotated data from eleven clinical centers in a centralized and an automatic way, in accordance with regulations such as GDPR. Involving and training healthcare professionals with experience in medical imaging, such as radiographers in the collection and annotation of imaging data, can be another solution to address the time-consuming data preparation and processing usually performed by radiologists.

The large variety of identified model architectures involving multiple parameters can be a double-edged sword at the conception stage. On the one hand, this allows more flexibility in the design and contributes to reinforcing some models (F3.2). On the other hand, this can also result in empirical model selection and parameter setting (B3.1) in the presence of ubiquitous technical and data constraints (B3.2 and B3.3). This contributes to the difficulty to choose the right model for the clinical target problem — fostering the need to gather all stakeholders (B3.4) to define common guidelines for the design and comparison of AI models [70]. Fortunately, the availability of code will continue to increase (~ 30% is publicly available or under request, Supplement Table S4) — supporting the reproducibility and transparency of published works.

### Clinical implementation

Despite several studies highlighting the potential of AI systems in terms of diagnostic performance (F2.1) and clinical workflow improvement (F2.2), only a few are properly implemented and used in clinical practice. In two recent systematic reviews on the use of AI in breast cancer screening programs, no prospective study for test accuracy was found [71]. This is a crucial point (B4.1) as a prospective study design is crucial to assess real clinical impact. Multi-center studies are also very important to assess the generalizability of developed AI systems, with different devices, acquisition protocols, and populations [3]. If data can be shared across institutions, validation on other data and imaging devices can be facilitated, and continuous update and quality control on AI systems would be possible.

Among the included studies, model transparency (B2.1) was an often-reported barrier. The black-box nature of modern AI algorithms is a major barrier to clinical implementation due to limited explanations for decisions, and the possible presence of bias — eroding the trust of clinicians and patients. Many methods to explain decisions of AI models are being investigated by the ML community, but these should be validated in clinical practice with end-users [72, 73]. Moving forward, it is imperative that future research focuses on the systematic development of more transparent AI algorithms using or creating explainability methods. Furthermore, these methods need to be evaluated to prevent potential biases.

These ethical aspects are closely related to legal concerns that are rising with the development of AI. At present, there are no international laws or consensus on the guidelines for the regulation of AI in medicine. However, many international supportive actions (e.g., WHO [74] and OCDE [75]) and national initiatives are being developed (e.g., USA FDA’s Software as Medical Device Action Plan [11] or EU’s “right to explanation” for patients [76]). Nevertheless, we strongly believe that achieving an international consensus for AI regulation is imperative, as opposed to relying solely on regional or national initiatives.

### Specificities of breast imaging modalities

Identified barriers and facilitators were globally encountered in each modality of clinical breast imaging (MG, DBT, US, or MRI); nevertheless, nuances related to the specificities of each modality were also identified. Compared to other modalities, US is more strongly operator-dependent due to the operator’s level of expertise, image quality appreciation, variability of acquisition (e.g., probe positioning, tissue compression, use of other imaging techniques like Doppler or elastography), and device parameters. This variability hinders the training and application of models [77–79]. In addition, careful attention is required when using common augmentation techniques as they may alter typical patterns of breast lesions (e.g., the presence of posterior acoustic shadow) [79]. Finally, embedded AI systems are often desired for real-time US investigation, introducing significant technical constraints given their low computing capabilities [54].

Even if AI methods developed for MG can be applied to DBT with transfer learning [80], DBT still presents some particularities related to the acquisition at multiple angles allowing the assessment of different depths of the breast [80]. This process is not standardized among clinical sites (number of images and angular range), enforcing the need for multi-center and multivendor studies [81].

Breast MRI generates a large amount of data, mainly due to the multi-parametric nature of this modality (multiple types of sequences), involving high dimensional data, e.g., DCE-MRI (dynamic contrast-enhanced MRI), 4D ultrafast sequences (series of 3D images over time with a high temporal resolution allowing visualization of contrast media uptake within 1 min), and diffusion sequences. The resulting series of 3D and 4D data are difficult to handle due to technical constraints (memory and computing resources), and the absence of off-the-shelf ML architectures to efficiently support and process the data (especially for 4D data). This often results in data transformation (such as dimensionality reduction) with the risk of losing information [10].

### Methodological choices with limitations and perspectives

Despite AI performance indicators being predominantly present in the surveyed literature, we purposely chose to not focus on performance metrics in the context of a scoping review. However, the dimension of performance was considered in the facilitators when a positive clinical impact was reported, since a well-performing AI solution will more likely be implemented or accepted in clinical practice.

In addition to works that explicitly mentioned barriers and/or facilitators of AI in breast imaging diagnosis, we decided to also include some papers that indirectly refer to barriers or facilitators based on the study design or author comments. Similarly, we included some publications not exclusively focusing on breast cancer as they were relevant in the context of breast cancer imaging and because they enriched the analysis of identified barriers and facilitators. The decision to include reviews, opinion articles, and original research also contributed to a more exhaustive scoping of the literature.

Most of the included papers originated from the USA, China, South Korea, India, and European countries (Fig. 2); thus, the results of this scoping review cannot reflect all the specificities of countries across the globe. For instance, depending on the difficulties of access or socio-economic constraints of some countries, some barriers may become more important [82] but facilitators may also appear, such as a greater demand for AI to counter a shortage of qualified professionals. This could pave the way for future work focusing on geographic, cultural, and socio-economic issues and potential discrepancies that could be related to AI introduction in breast cancer care.

### Authors’ opinion

There is still work to be done before AI systems are implemented sustainably in clinical practice. Regarding research, we believe it is important to promote transparency and reproducibility. Furthermore, it is important to acknowledge that AI models are susceptible to biases, particularly in cases where data are imbalanced or certain segments of the population are under-represented. To address this concern, it is recommended in the literature that research systematically includes demographic data (such as age, sex, ethnicity) and provides a comprehensive description of the measures taken to ensure data quality [68, 83]. This approach allows for the evaluation of biases within the models.

In practice, it appears urgent to establish methods for data collection and processing through the development of automated tools and active involvement of all health professionals engaged in breast imaging including radiographers, radiologists, and medical physicists. For instance, radiographers could assume a more significant role [19] in tasks such as data collection, data processing, protocol optimization, evaluation, clinical integration, and quality control for AI development and maintenance [84–86]. Another critical aspect to emphasize is that trust, from health professionals, patients, and also society in general, can impact the integration and adoption of AI in clinical settings. To address this barrier, it is crucial to focus on transparent AI algorithms with robust explainability methods and user-friendly AI interface systems during the conception stage. Additionally, more attention should be paid to the education of health professionals and patients about AI. This scoping review revealed a persistent lack of education among healthcare professionals, with insufficient integration of AI into their curricula despite recommendations [11, 87]. The acceptance of AI by patients is also a determining factor, with studies indicating that patient utilization of AI is more acceptable when under the continuous supervision of a physician [88]. This is understandable since physicians consider the patient’s complete medical history, while AI systems often focus solely on the imaging modality for which they are designed. Patients also harbor concerns regarding AI, such as preserving choice and autonomy, ensuring AI safety, and managing costs [89]. It is essential to address these concerns by implementing practices such as obtaining informed consent, establishing robust data privacy security measures, and ensuring that AI services are covered by health insurance. In conclusion, we strongly recommend the inclusion of AI training in professional education programs, accompanied by continuous training to keep up with the rapidly evolving techniques. Moreover, patients should be actively involved by receiving adequate information and being given the opportunity to participate in decisions regarding their care management and treatment. All stakeholders, including patients and healthcare professionals, should be actively engaged in the development of AI in healthcare.

## Conclusion

By identifying barriers and facilitators along with suggested solutions, this scoping review can provide valuable guidance to inform future research endeavors and support stakeholders in enhancing the design and implementation of AI for breast cancer detection in clinical practice. It highlighted the need to carefully conceive, deploy, and evaluate AI solutions in practice. Fortunately, most identified barriers had corresponding facilitators — showing that solutions are being explored to mitigate the current issues faced by clinical AI. There is little doubt that AI can improve breast cancer imaging, but a lot of coordinated effort among stakeholders will be required. In particular, health professionals involved in the production and consumption of medical images should be trained in AI principles and closely interact with AI systems.

### Supplementary Information

Below is the link to the electronic supplementary material.

Supplementary file1 (DOCX 390 KB)

## Abbreviations

AI: Artificial intelligence
AUC: Area under the curve
CAD: Computer-aided diagnosis/detection
CINHAL: Cumulative Index to Nursing and Allied Health Literature
CT: Computed tomography
DBT: Digital breast tomosynthesis
DCE: Dynamic contrast-enhanced
FDA: Food and Drug Administration
GDPR: General Data Protection Regulation
IEEE: Institute of Electrical and Electronics Engineers
MG: Digital mammography
ML: Machine learning
MRI: Magnetic resonance imaging
PACS: Picture Archiving and Communication System
PRISMA: Preferred Reporting Items for Systematic Reviews and Meta-Analyses
QA: Quality assurance
RIS: Radiology information systems
US: Ultrasound

## Declarations

### Guarantor

The scientific guarantor of this publication is Prof. Jérôme Schmid.

### Statistics and biometry

No complex statistical methods were necessary for this paper.

### Methodology

• Scoping review
