# Supplementary material for: Barriers and facilitators of artificial intelligence conception and implementation for breast imaging diagnosis in clinical practice: a scoping review
Source: Eur Radiol. 2023 Sep 2;34(3):2096–109. doi: 10.1007/s00330-023-10181-6 (PMC10873444; doi:10.1007/s00330-023-10181-6)
Supplement: Supplementary file 1 — Supplementary file1 (DOCX 390 KB) [file 330_2023_10181_MOESM1_ESM.docx]

# **Supplement S1:** search strategies

**Medline via PubMed**

("Breast Neoplasms"[Mesh] OR (("Breast"[Mesh]) OR (Breast[Title/Abstract] OR "Mammary Gland*"[Title/Abstract])) **AND** ("Neoplasms"[Mesh] OR Neoplasm*[Title/Abstract] OR Cancer*[Title/Abstract] OR Carcinoma[Title/Abstract] OR Oncolog*[Title/Abstract] OR Tumour*[Title/Abstract] OR Tumor*[Title/Abstract] OR Malignan*[Title/Abstract])) **AND** ("Diagnostic Imaging"[Mesh] OR (("Mass Screening"[Mesh] OR "Diagnosis"[Mesh] OR "Missed Diagnosis"[Mesh] OR "Diagnosis"[Title/Abstract] OR "Diagnostic"[Title/Abstract] OR "Detection*"[Title/Abstract] OR "Screening"[Title/Abstract]) **AND** ("Tomography Scanners, X Ray Computed"[MeSH Terms] OR "Mammogra*"[Title/Abstract] OR "Ultrasound*"[Title/Abstract] OR "CT Scan"[Title/Abstract] OR "Tomograph*"[Title/Abstract] OR "Tomosynthesis"[Title/Abstract] OR "Magnetic Resonance"[Title/Abstract] OR "Imaging"[Title/Abstract] OR "MRI"[Title/Abstract]))) **AND** ("Artificial Intelligence"[Mesh] OR "Artificial intelligence"[Title/Abstract] OR "Deep learning"[Title/Abstract] OR "Machine Learning"[Title/Abstract] OR "Neural network*"[Title/Abstract] OR "Computer assisted"[Title/Abstract] OR "Computer Aided Diagnosis"[Title/Abstract] OR "Computational Intelligence"[Title/Abstract] OR "Machine Intelligence"[Title/Abstract]) **AND** ("Decision Support Systems, Clinical"[Mesh] OR "Clinical Decision-Making"[Mesh] OR clinical[Title/Abstract])

| Date | 28.04.2022 |
| --- | --- |
| Results | 732 |
| Results with filter (last 10 years) | 535 |

**Web of Science**

((TS=(Breast* OR "Mammary Glands")) **AND** TS=(Cancer* OR Carcinoma OR Tumor* OR Tumour* OR Neoplasm* OR Malignan* OR Oncolog*)) AND (TS=("Diagnostic imaging") OR (TS=(Diagnosis OR Detection* OR Screening)) **AND** TS=(Mammogra* OR Ultrasound* OR "CT scan*" OR Tomograph* OR Tomosynthesis OR "Magnetic resonance" OR Imaging OR MRI)) **AND** (TS=("Artificial intelligence" OR "Deep learning" OR "Machine Learning" OR "Neural network*" OR "Computer assisted" OR "Computer Aided Diagnosis" OR "Computational Intelligence" OR "Machine Intelligence")) **AND** (TS=(clinical))

| Date | 29.04.2022 |
| --- | --- |
| Results | 1076 |
| Results with filter (last 10 years) | 833 |

**Embase**

('breast cancer'/exp OR (('breast'/exp OR breast*:ti,ab,kw OR 'mammary glands':ti,ab,kw) **AND** ('neoplasm'/exp OR cancer*:ti,ab,kw OR carcinoma:ti,ab,kw OR tumor*:ti,ab,kw OR tumour*:ti,ab,kw OR neoplasm*:ti,ab,kw OR malignan*:ti,ab,kw OR oncolog*:ti,ab,kw))) AND ('diagnostic imaging'/exp OR 'diagnostic imaging equipment'/exp OR (('diagnosis'/exp OR 'mass screening'/exp OR detection*:ti,ab,kw OR screening:ti,ab,kw OR diagnosis:ti,ab,kw OR diagnostic:ti,ab,kw) **AND** (mammogra*:ti,ab,kw OR ultrasound*:ti,ab,kw OR 'ct scan*':ti,ab,kw OR tomograph*:ti,ab,kw OR tomosynthesis:ti,ab,kw OR 'magnetic resonance':ti,ab,kw OR 'imaging':ti,ab,kw OR mri:ti,ab,kw))) **AND** ('artificial intelligence'/exp OR 'artificial intelligence':ti,ab,kw OR 'deep learning':ti,ab,kw OR 'machine learning':ti,ab,kw OR 'neural network*':ti,ab,kw OR 'computer assisted':ti,ab,kw OR 'computer aided diagnosis':ti,ab,kw OR 'computational intelligence':ti,ab,kw OR 'machine intelligence':ti,ab,kw) **AND** ('clinical decision making'/exp OR 'clinical decision support system'/de OR clinical:ti,ab,kw)

| Date | 02.05.2022 |
| --- | --- |
| Results | 916 |
| Results with filter (last 10 years) | 750 |

**Cinahl**

((MH "Breast Neoplasms+") OR (((MH "Neoplasms+") OR TI (cancer* OR carcinoma OR tumor* OR tumour* OR neoplasm* OR malignan* OR oncolog*) OR AB (cancer* OR carcinoma OR tumor* OR tumour* OR neoplasm* OR malignan* OR oncolog*))) **AND** ((MH "Breast+") OR TI (breast* OR "mammary glands") OR AB (breast* OR "mammary glands")))) AND ((MH "Diagnostic Imaging+") OR (((MH "Diagnosis+") OR (MH "Health Screening+") OR TI (diagnosis OR diagnostic OR detection* OR screening ) OR AB ( diagnosis OR diagnostic OR detection* OR screening)) AND (TI (mammogra* OR ultrasound* OR "CT scan*" OR tomograph* OR tomosynthesis OR "magnetic resonance" OR imaging OR MRI) OR AB (mammogra* OR ultrasound* OR "CT scan*" OR tomograph* OR tomosynthesis OR "magnetic resonance" OR imaging OR MRI)))) **AND** ((MH "Artificial Intelligence+") OR TI ("artificial intelligence" OR "deep learning" OR "machine learning" OR "neural network*" OR "computer assisted" OR "computer aided diagnosis" OR "computational intelligence" OR "machine intelligence") OR AB ("artificial intelligence" OR "deep learning" OR "machine learning" OR "neural network*" OR "computer assisted" OR "computer aided diagnosis" OR "computational intelligence" OR "machine intelligence")) **AND** ((MH "Decision Making, Clinical+") OR (MH "Decision Support Systems, Clinical") OR TI clinical OR AB clinical)

| Date | 29.04.2022 |
| --- | --- |
| Results | 177 |
| Results with filter (last 10 years) | 143 |

**IEEE Xplore**

(((((("All Metadata":Breast OR "All Metadata":"Mammary Glands") **AND** ("All Metadata":Cancer OR "All Metadata":Carcinoma OR "All Metadata":Tumor OR "All Metadata":Tumour OR "All Metadata":Neoplasm OR "All Metadata":Malignan* OR "All Metadata":Oncolog*))) AND (("All Metadata":Diagnosis OR "All Metadata":Diagnostic OR "All Metadata":Detection OR "All Metadata":Screening) **AND** ("All Metadata":Mammogra* OR "All Metadata":Ultrasound OR "All Metadata":"CT scan*" OR "All Metadata":Tomograph* OR "All Metadata":Tomosynthesis OR "All Metadata":"Magnetic resonance" OR "All Metadata":Imaging OR "All Metadata":MRI))) **AND** (("All Metadata":"Artificial intelligence" OR "All Metadata":"Deep learning" OR "All Metadata":"Machine Learning" OR "All Metadata":"Neural network*" OR "All Metadata":"Computer assisted" OR "All Metadata":"Computer Aided Diagnosis" OR "All Metadata":"Computational Intelligence" OR "All Metadata":"Machine Intelligence"))) **AND** (("All Metadata":Clinical)))

| Date | 27.04.2022 |
| --- | --- |
| Results | 327 |
| Results with filter (last 10 years) | 220 |

**arXiv**

size: 100; include_cross_list: True; terms: **AND** all=Breast; **AND** all=Cancer* OR Carcinoma OR Tumor* OR Tumour* OR Neoplasm* OR Malignan* OR Oncolog*; **AND** all=Diagnostic OR Diagnosis OR Detection* OR Screening; ND all=Mammogra* OR Ultrasound* OR "CT scan*" OR Tomograph* OR Tomosynthesis OR "Magnetic resonance" OR Imaging OR MRI; AND all="Artificial intelligence" OR "Deep learning" OR "Machine Learning" OR "Neural network*" OR "Computer assisted" OR "Computer Aided Diagnosis" OR "Computational Intelligence" OR "Machine Intelligence"; **AND** all=clinical

| Date | 28.04.2022 |
| --- | --- |
| Results after filter (last 10 years) | 69 |
| Results after pre-selection* | 28 |

* *A pre-selection was made based on titles and abstracts to facilitate the extraction of articles which was more constraining in this database.*

# **Supplement Table S2:** Barriers distribution among included papers

| Authors (year) | B1.1 | B1.2 | B1.3 | B2.1 | B2.2 | B2.3 | B3.1 | B3.2 | B3.3 | B3.4 | B4.1 | B4.2 | B4.3 | B5.1 | B5.2 | B5.3 | B5.4 | B5.5 | B6.1 |
| --- | --- | --- | --- | --- | --- | --- | --- | --- | --- | --- | --- | --- | --- | --- | --- | --- | --- | --- | --- |
| Akkus et al. (2019) [1] | x |  |  | x |  |  |  |  |  |  |  |  |  |  |  |  |  |  |  |
| Ayer et al. (2013) [2] | x | x | x | x |  |  | x | x |  |  |  |  |  |  |  |  |  |  |  |
| Bahl (2020) [3] | x | x |  | x | x | x |  |  | x |  | x | x |  |  |  |  |  |  | x |
| Bai et al. (2021) [4] | x | x |  | x | x | x |  |  | x | x | x | x |  |  |  |  |  |  |  |
| Balkenende et al. (2022) [5] | x |  | x |  |  |  |  | x | x |  | x | x |  | x |  |  |  |  |  |
| Baltzer (2021) [6] | x |  |  |  |  |  |  |  |  | x | x |  | x |  |  |  |  | x |  |
| Batchu et al. (2021) [7] |  |  |  |  |  |  | x | x | x | x | x | x | x |  |  | x | x |  |  |
| Bennani-Baiti et al. (2020) [8] | x | x | x |  |  |  |  | x |  |  | x |  |  |  | x |  |  | x |  |
| Benndorf et al. (2015) [9] |  |  |  |  |  |  |  |  |  |  |  | x |  |  |  |  |  |  |  |
| Bi et al. (2019) [10] |  | x |  | x |  |  |  |  |  | x | x |  | x | x | x | x |  | x |  |
| Bitencourt et al. (2021) [11] | x |  |  |  |  |  | x |  |  |  | x | x |  |  |  |  |  | x |  |
| Calisto et al. (2021) [12] |  | x |  | x | x |  |  |  | x |  | x |  | x |  |  | x |  |  |  |
| Chan et al. (2020) [13] | x | x |  | x |  |  |  |  |  |  | x |  | x |  |  |  |  |  | x |
| Chang et al. (2022) [14] |  |  |  |  |  |  |  |  |  |  | x |  |  |  |  |  |  |  |  |
| Chaudhary et al. (2021) [15] | x | x |  | x |  |  |  |  |  |  |  |  |  |  | x |  |  |  |  |
| Cheung et al. (2021) [16] | x | x |  |  |  |  |  |  |  | x | x | x |  | x | x | x |  |  |  |
| Choy et al. (2018) [17] | x | x |  | x |  |  |  |  |  |  | x | x | x |  | x |  |  |  |  |
| Ciritsis et al. (2019) [18] |  |  |  | x | x |  |  |  | x |  |  |  |  |  |  |  |  |  |  |
| Cuocolo et al. (2020) [19] |  | x |  | x |  |  |  |  | x | x |  |  |  |  | x | x |  |  |  |
| Dai et al. (2021) [20] |  |  |  |  |  |  |  | x |  |  |  |  | x |  |  |  |  |  |  |
| Daimiel Naranjo et al. (2022) [21] |  |  |  |  |  |  |  |  |  |  |  |  |  |  |  |  |  |  |  |
| Davatzikos et al. (2019) [22] | x | x |  |  |  |  |  |  |  |  |  |  |  |  |  |  |  |  |  |

| **Authors (year)** | **B1.1** | **B1.2** | **B1.3** | **B2.1** | **B2.2** | **B2.3** | **B3.1** | **B3.2** | **B3.3** | **B3.4** | **B4.1** | **B4.2** | **B4.3** | **B5.1** | **B5.2** | **B5.3** | **B5.4** | **B5.5** | **B6.1** |
| --- | --- | --- | --- | --- | --- | --- | --- | --- | --- | --- | --- | --- | --- | --- | --- | --- | --- | --- | --- |
| Deshmukh et al. (2021) [23] | x | x |  | x | x | x | x |  | x | x |  |  |  |  | x |  | x |  |  |
| Du et al. (2022) [24] |  |  |  | x |  |  |  |  | x | x |  |  |  |  |  |  |  |  |  |
| Elbatel et al. (2022) [25] | x |  | x | x |  |  |  |  |  |  |  | x |  |  |  |  |  |  |  |
| Elhakim et al. (2020) [26] |  |  |  |  |  |  |  |  |  | x | x | x | x |  | x |  | x |  |  |
| Fazal et al. (2018) [27] |  |  |  | x | x |  |  |  |  |  |  | x |  | x |  |  |  |  |  |
| Freeman et al. (2021) [28] |  |  |  | x |  |  |  |  |  | x | x | x |  |  |  | x |  |  |  |
| Fuchsjäger, (2019) [29] | x |  |  |  |  |  |  |  |  |  | x | x |  | x | x | x |  |  |  |
| Fujioka et al. (2020) [30] |  |  |  | x |  |  |  |  | x |  |  | x |  | x |  |  |  |  |  |
| Gao et al. (2019) [31] | x | x | x | x |  |  |  |  |  | x |  | x |  | x | x |  | x |  |  |
| Gardezi et al. (2019) [32] | x | x | x |  |  |  | x |  |  |  |  | x |  |  |  |  |  | x |  |
| Geras et al. (2019) [33] | x |  |  | x |  |  | x |  | x |  |  |  |  | x |  | x |  | x |  |
| Grimm (2021) [34] | x |  |  |  |  | x | x |  | x |  |  | x |  | x |  | x |  |  |  |
| Halim et al. (2021) [35] | x | x | x |  |  |  |  |  |  |  |  |  |  |  |  | x |  |  |  |
| Hameed et al. (2021) [36] | x |  |  |  |  |  | x |  |  |  |  |  |  |  |  |  |  |  |  |
| Han et al. (2017) [37] | x |  |  |  |  |  |  |  |  |  |  |  |  |  |  |  |  |  |  |
| Harvey et al. (2019) [38] |  |  |  |  |  |  |  | x |  | x | x |  |  |  |  |  |  |  |  |
| Heller et al. (2020) [39] |  |  |  |  |  |  |  |  |  |  | x |  |  |  |  |  |  |  |  |
| Hou et al. (2021) [40] | x | x |  |  |  |  |  |  |  |  |  |  |  |  |  |  |  |  |  |
| Houssami et al. (2019) [41] | x | x |  |  | x | x |  |  |  |  | x | x |  | x | x | x | x |  |  |
| Hu et al. (2021) [42] | x | x | x | x |  |  |  |  |  |  |  |  |  |  |  |  |  |  | x |
| Huang et al. (2020) [43] |  |  |  | x |  |  | x |  |  | x |  |  |  |  | x | x |  |  |  |
| Jiménez-Sánchez et al. (2021) [44] |  |  | x |  |  |  |  |  |  |  |  | x |  |  | x |  |  |  |  |
| Johansson et al. (2021) [45] |  |  |  |  |  |  |  |  |  |  |  | x |  |  |  |  |  |  |  |
| Kim et al. (2018) [46] |  |  |  | x |  |  |  |  |  |  |  |  |  |  |  |  |  |  |  |
| **Authors (year)** | **B1.1** | **B1.2** | **B1.3** | **B2.1** | **B2.2** | **B2.3** | **B3.1** | **B3.2** | **B3.3** | **B3.4** | **B4.1** | **B4.2** | **B4.3** | **B5.1** | **B5.2** | **B5.3** | **B5.4** | **B5.5** | **B6.1** |
| Kim et al. (2019) [47] |  |  |  | x |  |  |  | x | x |  |  | x |  |  |  |  |  |  |  |
| Kim et al. (2021) [48] | x | x |  | x |  |  |  |  |  |  | x | x |  |  |  |  |  |  |  |
| Komatsu et al. (2021) [49] | x | x |  | x |  |  |  |  |  | x | x | x |  |  | x | x |  |  |  |
| Kyono et al. (2018) [50] |  |  |  | x |  |  |  |  | x |  |  |  |  |  |  |  |  |  |  |
| Lamb et al. (2022) [51] |  |  | x | x | x | x |  | x |  | x | x | x |  | x | x | x |  |  |  |
| Lassau et al. (2019) [52] |  |  |  |  |  |  |  |  |  | x |  |  |  |  |  |  |  |  | x |
| Lassau et al. (2021) [53] | x | x |  |  |  |  |  |  | x | x |  |  |  |  |  |  |  |  |  |
| Le et al. (2019) [54] | x | x |  | x | x |  | x | x | x |  | x | x | x |  |  |  |  |  | x |
| Lee et al. (2020) [55] | x |  |  | x |  |  | x |  |  | x | x | x |  |  |  |  |  |  |  |
| Lee et al. (2020) [56] | x |  |  | x |  |  |  |  |  | x | x | x | x | x | x | x |  |  |  |
| Lei et al. (2021) [57] | x | x |  |  |  |  |  | x |  |  |  |  |  |  |  |  |  |  |  |
| Li et al. (2021) [58] | x |  |  |  |  |  |  | x |  | x |  |  | x |  |  |  |  |  |  |
| Lyu et al. (2022) [59] |  |  |  |  |  |  |  |  |  |  |  |  |  |  |  |  |  |  |  |
| Makino et al. (2020) [60] |  |  |  | x | x |  |  |  |  |  |  |  |  |  |  |  |  |  |  |
| Mann et al. (2020) [61] |  |  |  |  |  |  |  |  |  |  | x |  |  |  |  |  |  |  |  |
| Maqsood et al. (2022) [62] | x |  |  |  |  |  |  | x |  |  |  |  |  |  |  |  |  |  |  |
| Massat et al. (2019) [63] |  |  |  |  |  |  |  |  |  |  |  |  |  |  |  |  |  |  |  |
| Masud et al. (2019) [64] |  |  |  |  | x |  |  |  |  |  |  |  |  |  |  |  |  | x |  |
| Meyer-Bäse et al. (2020) [65] | x | x |  | x |  |  |  | x | x |  |  | x |  |  |  |  |  |  |  |
| Morgan et al. (2021) [66] |  |  |  | x | x | x |  |  |  |  | x | x |  |  |  |  |  |  | x |
| Neri et al. (2019) [67] | x | x |  |  |  |  |  |  |  |  |  |  |  | x | x | x |  |  |  |
| Nishikawa et al. (2014) [68] |  |  |  |  |  |  |  | x |  |  | x |  |  |  |  |  |  |  |  |
| Ou et al. (2021) [69] | x | x |  | x | x | x |  |  |  |  | x | x |  | x |  | x | x |  |  |
| Panayides et al. (2020) [70] | x | x | x | x |  |  |  |  | x |  |  | x |  |  | x |  |  |  |  |
| Pang et al. (2020) [71] | x | x |  | x |  |  |  |  |  |  |  | x | x |  |  |  |  |  |  |
| **Authors (year)** | **B1.1** | **B1.2** | **B1.3** | **B2.1** | **B2.2** | **B2.3** | **B3.1** | **B3.2** | **B3.3** | **B3.4** | **B4.1** | **B4.2** | **B4.3** | **B5.1** | **B5.2** | **B5.3** | **B5.4** | **B5.5** | **B6.1** |
| Pedemonte et al. (2022) [72] |  |  |  |  |  |  |  |  |  |  |  | x |  |  |  |  |  |  |  |
| Pesapane et al. (2021) [73] | x | x | x | x |  |  |  |  | x | x |  | x |  |  |  |  |  |  |  |
| Qian et al. (2021) [74] | x |  |  | x | x |  |  |  |  |  | x |  |  |  |  |  |  |  |  |
| Qiao et al. (2022) [75] | x |  |  |  |  |  |  |  | x |  |  |  |  |  |  |  |  |  |  |
| Reardon, (2019) [76] | x | x |  | x |  |  |  |  |  |  | x | x | x | x |  |  |  |  |  |
| Rizzi et al. (2013) [77] |  | x |  |  |  |  |  |  |  |  |  | x |  |  |  |  |  |  |  |
| Rodriguez-Ruiz et al. (2019) [78] |  |  |  |  |  |  |  |  |  |  | x |  |  |  |  |  |  |  |  |
| Rodriguez-Ruiz et al. (2019) [79] |  |  |  |  | x |  |  |  |  |  | x | x |  | x | x |  |  |  |  |
| Sahiner et al. (2019) [80] | x | x |  | x |  |  |  |  |  | x |  | x |  |  |  |  |  |  |  |
| Samala et al. (2021) [81] | x |  |  |  |  |  | x |  |  |  | x | x |  |  |  |  |  |  |  |
| Satake et al. (2022) [82] | x | x |  |  |  |  |  |  |  | x | x | x |  | x |  | x |  |  |  |
| Sato et al. (2014) [83] |  |  |  |  |  |  |  |  |  |  |  |  |  |  |  |  |  |  |  |
| Schaffter et al. (2020) [84] |  |  |  |  |  |  |  |  | x |  |  |  |  |  |  |  |  |  |  |
| Sechopoulos et al. (2021) [85] | x | x |  |  |  |  |  |  |  |  | x |  | x | x |  | x |  |  |  |
| Shan et al. (2021) [86] | x | x |  |  |  |  |  |  |  |  |  |  |  |  |  |  |  |  |  |
| Shastry et al. (2022) [87] |  |  |  |  |  |  |  |  |  |  |  |  |  |  |  |  |  | x |  |
| Shen et al. (2021) [88] | x | x |  | x |  |  |  |  |  |  |  |  |  |  | x |  | x |  |  |
| Shoshan et al. (2022) [89] |  |  |  |  | x |  |  |  |  |  | x |  |  |  |  |  |  |  |  |
| Song et al. (2016) [90] |  | x |  | x |  |  |  |  | x |  |  |  |  |  |  |  |  |  |  |
| Subuhana et al. (2021) [91] | x | x |  | x |  |  |  |  |  |  |  | x |  |  |  |  |  |  |  |
| Tadavarthi et al. (2020) [92] |  |  |  | x |  |  |  |  |  | x |  | x |  | x | x |  | x | x | x |
| Tagliafico et al. (2020) [93] | x | x | x |  |  |  |  |  |  |  | x | x | x |  |  |  |  |  | x |
| Tartar et al. (2021) [94] |  |  |  |  |  |  |  |  |  |  |  |  |  |  |  |  |  |  |  |
| **Authors (year)** | **B1.1** | **B1.2** | **B1.3** | **B2.1** | **B2.2** | **B2.3** | **B3.1** | **B3.2** | **B3.3** | **B3.4** | **B4.1** | **B4.2** | **B4.3** | **B5.1** | **B5.2** | **B5.3** | **B5.4** | **B5.5** | **B6.1** |
| Tasdemir et al. (2020) [95] |  | x |  |  |  |  |  |  |  | x |  | x |  |  |  |  |  |  |  |
| Thomassin-Naggara et al. (2019) [96] | x |  |  |  |  |  |  |  |  |  | x | x |  |  |  |  |  | x |  |
| Trivizakis et al. (2020) [97] | x | x |  | x | x | x |  |  | x | x |  | x |  | x |  |  |  |  |  |
| Vobugari et al. (2022) [98] | x | x |  | x |  |  |  |  |  | x |  | x |  | x | x | x |  |  | x |
| Wang et al. (2020) [99] | x |  |  | x |  |  | x |  | x |  | x | x |  |  | x |  |  |  |  |
| Wang et al. (2020) [100] | x | x |  |  |  |  | x | x | x |  |  | x |  |  |  |  |  |  |  |
| Wichmann et al. (2020) [101] |  | x |  | x | x | x |  | x |  | x | x | x | x |  | x | x | x | x |  |
| Yu et al. (2021) [102] |  |  |  |  |  |  |  |  | x |  |  | x |  |  |  |  |  |  |  |
| Zhang et al. (2019) [103] | x | x | x | x | x | x | x |  |  |  | x | x | x | x |  | x |  |  |  |
| Zhang et al. (2021) [104] |  |  |  |  |  |  |  |  | x |  |  |  |  |  |  |  |  |  |  |
| Zhang et al. (2022) [105] | x | x |  | x |  |  |  |  |  | x | x | x |  |  |  |  |  |  |  |
| Zhou et al. (2021) [106] |  |  |  |  |  |  |  |  |  |  |  |  |  |  |  |  | x |  |  |
| Zou et al. (2019) [107] | x | x | x | x |  |  | x | x | x |  | x | x |  |  |  |  |  |  |  |
| Total | 60 | 48 | 15 | 51 | 19 | 11 | 16 | 17 | 28 | 29 | 44 | 54 | 17 | 22 | 24 | 22 | 10 | 11 | 9 |

B1.1: Data size and variety, B1.2: Data quality and data processing, B1.3: Data sharing, B2.1: Model transparency, B2.2: Clinician trust, B2.3: Patient trust, B3.1: Model architecture, B3.2: Technical constraints, B3.3: Multivariable data, B3.4: Involvement of stakeholders, B4.1: Meaningful clinical evaluation, B4.2: Data variability, B4.3: Quality assurance, B5.1: Liability , B5.2: Law and policies, B5.3: Fair AI, B5.4: Cybersecurity , B5.5: Economic issues, B6.1: Education

# **Supplement Table S3:** Facilitators distribution among included papers

| Authors (year) | F1.1 | F1.2 | F2.1 | F2.2 | F3.1 | F3.2 | F4.1 | F4.2 | F5.1 |
| --- | --- | --- | --- | --- | --- | --- | --- | --- | --- |
| Akkus et al. (2019) [1] |  | x |  |  | x |  |  |  |  |
| Ayer et al. (2013) [2] |  |  |  |  |  | x |  |  |  |
| Bahl (2020) [3] |  |  | x | x |  |  |  |  |  |
| Bai et al. (2021) [4] | x |  |  | x |  |  |  |  |  |
| Balkenende et al. (2022) [5] |  | x |  |  | x |  |  |  | x |
| Baltzer (2021) [6] |  |  | x |  |  | x |  |  |  |
| Batchu et al. (2021) [7] |  |  | x | x |  | x |  |  |  |
| Bennani-Baiti et al. (2020) [8] | x |  | x |  |  |  |  | x |  |
| Benndorf et al. (2015) [9] |  |  |  |  |  |  |  |  |  |
| Bi et al. (2019) [10] |  | x | x | x | x |  |  |  |  |
| Bitencourt et al. (2021) [11] | x |  |  | x |  |  |  |  |  |
| Calisto et al. (2021) [12] |  |  | x | x |  |  |  |  |  |
| Chan et al. (2020) [13] |  | x |  | x |  |  |  |  |  |
| Chang et al. (2022) [14] |  |  |  |  |  |  |  |  |  |
| Chaudhary et al. (2021) [15] |  |  |  | x |  |  |  |  |  |
| Cheung et al. (2021) [16] |  | x | x | x | x |  |  |  |  |
| Choy et al. (2018) [17] |  |  | x |  |  |  |  |  |  |
| Ciritsis et al. (2019) [18] |  |  | x |  |  |  |  |  |  |
| Cuocolo et al. (2020) [19] |  |  |  | x |  | x |  |  |  |
| Dai et al. (2021) [20] |  |  |  | x | x |  |  |  |  |
| Daimiel Naranjo et al. (2022) [21] |  |  | x | x | x |  |  |  |  |
| Davatzikos et al. (2019) [22] |  |  |  |  | x |  |  |  |  |
| Deshmukh et al. (2021) [23] |  | x | x |  | x |  |  |  |  |
| Du et al. (2022) [24] |  |  | x | x |  |  |  |  |  |
| Elbatel et al. (2022) [25] |  | x | x |  |  |  |  |  |  |
| Elhakim et al. (2020) [26] |  |  | x | x |  |  |  |  |  |
| Fazal et al. (2018) [27] |  | x | x | x | x |  |  |  |  |
| Freeman et al. (2021) [28] |  |  |  | x |  |  |  |  |  |
| Fuchsjäger, (2019) [29] |  |  |  | x |  |  |  |  |  |
| Fujioka et al. (2020) [30] |  |  | x | x | x |  |  |  |  |
| Gao et al. (2019) [31] | x | x | x | x |  |  |  |  |  |
| Gardezi et al. (2019) [32] | x | x |  |  |  |  |  |  |  |
| Geras et al. (2019) [33] |  | x | x | x | x |  |  |  |  |
| Grimm (2021) [34] |  |  |  |  | x |  | x |  |  |
| Halim et al. (2021) [35] |  |  | x |  |  |  |  |  |  |
| Hameed et al. (2021) [36] |  |  | x |  |  | x |  |  |  |
| Han et al. (2017) [37] |  |  | x | x |  |  |  |  |  |
| Harvey et al. (2019) [38] | x | x |  | x |  | x |  |  | x |
| Heller et al. (2020) [39] |  |  | x |  |  |  |  |  |  |
| Hou et al. (2021) [40] |  |  | x | x |  |  |  |  |  |
| **Authors (year)** | **F1.1** | **F1.2** | **F2.1** | **F2.2** | **F3.1** | **F3.2** | **F4.1** | **F4.2** | **F5.1** |
| Houssami et al. (2019) [41] |  |  | x | x | x |  |  |  |  |
| Hu et al. (2021) [42] |  |  | x | x | x | x |  |  |  |
| Huang et al. (2020) [43] |  |  | x |  |  | x |  |  | x |
| Jiménez-Sánchez et al. (2021) [44] |  | x |  |  |  |  |  |  |  |
| Johansson et al. (2021) [45] |  |  | x | x |  |  |  |  |  |
| Kim et al. (2018) [46] |  |  | x |  |  |  |  |  |  |
| Kim et al. (2019) [47] |  |  | x | x |  |  |  |  |  |
| Kim et al. (2021) [48] |  | x |  |  |  |  |  |  |  |
| Komatsu et al. (2021) [49] |  |  | x | x | x |  | x |  | x |
| Kyono et al. (2018) [50] |  |  | x | x |  |  |  |  |  |
| Lamb et al. (2022) [51] |  |  |  | x |  |  |  |  |  |
| Lassau et al. (2019) [52] | x |  |  |  |  |  |  | x |  |
| Lassau et al. (2021) [53] |  |  |  |  |  |  |  | x |  |
| Le et al. (2019) [54] | x |  |  | x | x |  |  | x |  |
| Lee et al. (2020) [55] | x | x |  |  |  | x |  |  |  |
| Lee et al. (2020) [56] | x |  |  |  |  |  |  | x |  |
| Lei et al. (2021) [57] |  |  | x | x |  |  |  |  |  |
| Li et al. (2021) [58] |  |  |  |  |  |  |  |  |  |
| Lyu et al. (2022) [59] |  |  | x | x |  |  |  |  |  |
| Makino et al. (2020) [60] |  |  |  |  |  |  |  |  |  |
| Mann et al. (2020) [61] |  |  | x | x |  |  |  |  |  |
| Maqsood et al. (2022) [62] |  |  | x | x |  |  |  |  |  |
| Massat et al. (2019) [63] |  |  | x | x |  |  |  |  |  |
| Masud et al. (2019) [64] |  |  |  | x |  |  |  |  |  |
| Meyer-Bäse et al. (2020) [65] |  | x |  | x | x |  |  |  |  |
| Morgan et al. (2021) [66] |  |  | x | x |  |  |  |  |  |
| Neri et al. (2019) [67] |  |  |  |  |  |  |  |  |  |
| Nishikawa et al. (2014) [68] |  |  | x |  |  |  |  |  |  |
| Ou et al. (2021) [69] |  |  | x | x |  |  |  |  |  |
| Panayides et al. (2020) [70] |  | x | x | x |  |  |  |  |  |
| Pang et al. (2020) [71] |  |  |  |  | x |  |  |  |  |
| Pedemonte et al. (2022) [72] |  |  | x | x |  |  |  |  |  |
| Pesapane et al. (2021) [73] |  |  |  |  |  |  |  |  |  |
| Qian et al. (2021) [74] |  | x |  |  |  |  |  |  |  |
| Qiao et al. (2022) [75] |  |  |  |  | x |  |  |  |  |
| Reardon, (2019) [76] |  |  |  | x |  |  |  |  |  |
| Rizzi et al. (2013) [77] |  |  |  |  |  |  |  |  |  |
| Rodriguez-Ruiz et al. (2019) [78] |  |  | x | x |  |  |  |  |  |
| Rodriguez-Ruiz et al. (2019) [79] |  |  | x | x |  |  |  |  |  |
| Sahiner et al. (2019) [80] | x | x |  |  |  | x |  |  |  |
| Samala et al. (2021) [81] |  | x |  |  |  |  |  |  |  |
| **Authors (year)** | **F1.1** | **F1.2** | **F2.1** | **F2.2** | **F3.1** | **F3.2** | **F4.1** | **F4.2** | **F5.1** |
| Satake et al. (2022) [82] |  |  |  |  | x | x |  |  |  |
| Sato et al. (2014) [83] |  |  | x | x |  |  |  |  |  |
| Schaffter et al. (2020) [84] | x |  | x |  |  |  |  | x |  |
| Sechopoulos et al. (2021) [85] |  | x | x | x |  |  |  |  |  |
| Shan et al. (2021) [86] |  |  |  | x | x |  |  |  |  |
| Shastry et al. (2022) [87] |  |  |  |  |  |  |  |  |  |
| Shen et al. (2021) [88] |  | x | x | x |  |  |  |  |  |
| Shoshan et al. (2022) [89] |  |  | x | x |  |  |  |  |  |
| Song et al. (2016) [90] |  |  |  | x | x |  |  |  |  |
| Subuhana et al. (2021) [91] | x |  |  |  |  |  |  |  |  |
| Tadavarthi et al. (2020) [92] |  |  |  |  |  |  |  |  |  |
| Tagliafico et al. (2020) [93] |  |  |  | x |  |  |  |  |  |
| Tartar et al. (2021) [94] |  |  |  | x |  |  |  |  |  |
| Tasdemir et al. (2020) [95] |  |  |  |  |  |  |  |  |  |
| Thomassin-Naggara et al. (2019) [96] |  |  | x |  |  |  |  |  | x |
| Trivizakis et al. (2020) [97] | x |  | x |  | x |  |  |  |  |
| Vobugari et al. (2022) [98] |  |  | x | x |  |  |  |  |  |
| Wang et al. (2020) [99] |  |  |  |  |  |  |  |  |  |
| Wang et al. (2020) [100] |  | x | x | x |  |  |  |  |  |
| Wichmann et al. (2020) [101] |  | x |  | x |  |  | x |  |  |
| Yu et al. (2021) [102] |  |  |  | x |  |  |  |  |  |
| Zhang et al. (2019) [103] |  | x |  |  |  |  |  |  |  |
| Zhang et al. (2021) [104] |  |  | x | x | x |  |  |  |  |
| Zhang et al. (2022) [105] |  | x |  |  |  |  |  |  |  |
| Zhou et al. (2021) [106] |  |  |  |  |  |  |  |  |  |
| Zou et al. (2019) [107] | x | x |  |  |  |  |  |  |  |
| Total | 15 | 27 | 53 | 58 | 24 | 11 | 3 | 6 | 5 |

F1.1: Datasets initiatives, F1.2: Algorithmic approaches to address data barriers, F2.1: Diagnostic performance, F2.2: Clinical workflow, F3.1: Multivariable data F3.2: Numerous algorithms, F4.1: Increased accessibility of AI, F4.2: Benchmarking of AI approaches, F5.1: AI for education

# **Supplement Table S4:** Information and characteristics of included papers

| Authors (year) | Origin | Language* | Paper type | Data availability | Code availability | Imaging modality |
| --- | --- | --- | --- | --- | --- | --- |
| Akkus et al. (2019) [1] | USA | English | review | NA | NA | US |
| Ayer et al. (2013) [2] | USA | English | review | NA | NA | MG |
| Bahl (2020) [3] | USA | English | review | NA | NA | MG |
| Bai et al. (2021) [4] | USA | English | review | NA | NA | DBT |
| Balkenende et al. (2022) [5] | Netherlands | English | review | NA | NA | ALL |
| Baltzer (2021) [6] | Austria | German | opinion article | NA | NA | ALL |
| Batchu et al. (2021) [7] | USA | English | review | NA | NA | MG |
| Bennani-Baiti et al. (2020) [8] | Austria | German | review | NA | NA | ALL |
| Benndorf et al. (2015) [9] | Germany | English | original research | NO | NO | MG |
| Bi et al. (2019) [10] | USA | English | review | NA | NA | ALL |
| Bitencourt et al. (2021) [11] | Brazil | English | review | NA | NA | ALL |
| Calisto et al. (2021) [12] | Portugal | English | original research | NO | NO | ALL |
| Chan et al. (2020) [13] | USA | English | review | NA | NA | ALL |
| Chang et al. (2022) [14] | South Korea | English | original research | NO | NO | MG |
| Chaudhary et al. (2021) [15] | India | English | review | NA | NA | MG |
| Cheung et al. (2021) [16] | Canada | English | review | NA | NA | MG |
| Choy et al. (2018) [17] | USA | English | review | NA | NA | ALL |
| Ciritsis et al. (2019) [18] | Switzerland | English | original research | NO | NO | US |
| Cuocolo et al. (2020) [19] | Italy | English | review | NA | NA | ALL |
| Dai et al. (2021) [20] | China | English | original research | NO | NO | US |
| Daimiel Naranjo et al. (2022) [21] | USA | English | original research | UR | YES | IRM |
| Davatzikos et al. (2019) [22] | USA | English | review | NA | NA | IRM |
| Deshmukh et al. (2021) [23] | India | English | review | NA | NA | ALL |
| Du et al. (2022) [24] | China | English | original research | UR | NO | US |
| Elbatel et al. (2022) [25] | Spain | English | review | NA | NA | MG |
| Elhakim et al. (2020) [26] | Denmark | Danish | review | NA | NA | MG |
| Fazal et al. (2018) [27] | England | English | review | NA | NA | MG |
| Freeman et al. (2021) [28] | England | English | systematic review | NA | NA | MG |
| Fuchsjäger, (2019) [29] | Austria | English | editorial comment | NA | NA | ALL |
| Fujioka et al. (2020) [30] | Japan | English | review | NA | NA | US |
| Gao et al. (2019) [31] | USA | English | review | NA | NA | MG+DBT |
| Gardezi et al. (2019) [32] | China | English | systematic review | NA | NA | MG |
| Geras et al. (2019) [33] | Netherlands | English | review | NA | NA | MG+DBT |
| **Authors (year)** | **Location** | **Language*** | **Paper type** | **Data availability** | **Code availability** | **Imaging modality** |
| Grimm (2021) [34] | USA | English | review | NA | NA | ALL |
| Halim et al. (2021) [35] | Malaysia | English | review | NA | NA | ALL |
| Hameed et al. (2021) [36] | India | English | review | NA | NA | MRI |
| Han et al. (2017) [37] | South Korea | English | original research | NO | NO | US |
| Harvey et al. (2019) [38] | England | English | review | NA | NA | MG+DBT |
| Heller et al. (2020) [39] | USA | English | original research | NO | NO | US |
| Hou et al. (2021) [40] | USA | English | original research | NO | YES | MG |
| Houssami et al. (2019) [41] | Australia | English | scoping review | NA | NA | MG |
| Hu et al. (2021) [42] | USA | English | review | NA | NA | ALL |
| Huang et al. (2020) [43] | China | English | review | NA | NA | ALL |
| Jiménez-Sánchez et al. (2021) [44] | Spain | English | original research | NO | YES | MG |
| Johansson et al. (2021) [45] | Sweden | English | original research | NO | NA | MG |
| Kim et al. (2018) [46] | South Korea | English | original research | NO | NA | MG |
| Kim et al. (2019) [47] | South Korea | English | original research | NO | NO | US |
| Kim et al. (2021) [48] | South Korea | English | review | NA | NA | US |
| Komatsu et al. (2021) [49] | Japan | English | review | NA | NA | US |
| Kyono et al. (2018) [50] | USA | English | original research | NO | NO | MG |
| Lamb et al. (2022) [51] | USA | English | review | NA | NA | MG |
| Lassau et al. (2019) [52] | France | English | original research | NO | NO | US+CT+ MRI |
| Lassau et al. (2021) [53] | France | English | original research | NO | NO | CT+US |
| Le et al. (2019) [54] | England | English | review | NA | NA | ALL |
| Lee et al. (2020) [55] | South Korea | English | review | NA | NA | ALL |
| Lee et al. (2020) [56] | USA | English | original research | NO | NO | MG |
| Lei et al. (2021) [57] | China | English | review | NA | NA | ALL |
| Li et al. (2021) [58] | China | English | original research | UR | NO | MG |
| Lyu et al. (2022) [59] | China | English | original research | NO | NA | US |
| Makino et al. (2020) [60] | USA | English | original research | YES | YES | MG |
| Mann et al. (2020) [61] | Netherlands | English | review | NA | NA | MRI |
| Maqsood et al. (2022) [62] | Lithuania | English | original research | UR | UR | MG |
| Massat et al. (2019) [63] | USA | English | opinion article | NA | NA | ALL |
| Masud et al. (2019) [64] | Canada | English | scoping review | NA | NA | ALL |
| Meyer-Bäse et al. (2020) [65] | USA | English | review | NA | NA | MRI |
| Morgan et al. (2021) [66] | USA | English | review | NA | NA | MG |
| Neri et al. (2019) [67] | Austria | English | review | NA | NA | ALL |
| Nishikawa et al. (2014) [68] | USA | English | opinion article | NA | NA | MG |
| **Authors (year)** | **Location** | **Language*** | **Paper type** | **Data availability** | **Code availability** | **Imaging modality** |
| Ou et al. (2021) [69] | USA | English | review | NA | NA | ALL |
| Panayides et al. (2020) [70] | Cyprus | English | review | NA | NA | ALL |
| Pang et al. (2020) [71] | Malaysia | English | review | NA | NA | ALL |
| Pedemonte et al. (2022) [72] | USA | English | original research | NO | NO | MG |
| Pesapane et al. (2021) [73] | Italy | English | review | NA | NA | ALL |
| Qian et al. (2021) [74] | China | English | original research | NO | UR | US |
| Qiao et al. (2022) [75] | China | English | original research | NO | NO | MRI+US |
| Reardon, (2019) [76] | USA | English | opinion article | NA | NA | ALL |
| Rizzi et al. (2013) [77] | Italy | English | original research | NO | NO | MG |
| Rodriguez-Ruiz et al. (2019) [78] | Netherlands | English | original research | NO | NO | MG |
| Rodriguez-Ruiz et al. (2019) [79] | Netherlands | English | original research | NO | NA | MG |
| Sahiner et al. (2019) [80] | USA | English | review | NA | NA | ALL |
| Samala et al. (2021) [81] | USA | English | original research | NO | NO | MG |
| Satake et al. (2022) [82] | Japan | English | review | NA | NA | MRI |
| Sato et al. (2014) [83] | Japan | English | original research | NO | NO | MG |
| Schaffter et al. (2020) [84] | USA | English | original research | NO | NO | MG |
| Sechopoulos et al. (2021) [85] | Netherlands | English | review | NA | NA | MG+DBT |
| Shan et al. (2021) [86] | China | English | review | NA | NA | US |
| Shastry et al. (2022) [87] | India | English | review | NA | NA | ALL |
| Shen et al. (2021) [88] | China | English | review | NA | NA | US |
| Shoshan et al. (2022) [89] | Israel | English | original research | NO | YES | DBT |
| Song et al. (2016) [90] | USA | English | original research | NO | NO | MG |
| Subuhana et al. (2021) [91] | India | English | review | NA | NA | ALL |
| Tadavarthi et al. (2020) [92] | USA | English | review | NA | NA | ALL |
| Tagliafico et al. (2020) [93] | Italy | English | review | NA | NA | ALL |
| Tartar et al. (2021) [94] | USA | English | case study | NA | NA | MG |
| Tasdemir et al. (2020) [95] | Turkey | English | review | NA | NA | MG |
| Thomassin-Naggara et al. (2019) [96] | France | English | review | NA | NA | MG |
| Trivizakis et al. (2020) [97] | Greece | English | review | NA | NA | ALL |
| Vobugari et al. (2022) [98] | USA | English | review | NA | NA | ALL |
| Wang et al. (2020) [99] | USA | English | original research | NO | NO | MG |
| Wang et al. (2020) [100] | China | English | review | NA | NA | ALL |
| **Authors (year)** | **Location** | **Language*** | **Paper type** | **Data availability** | **Code availability** | **Imaging modality** |
| Wichmann et al. (2020) [101] | Germany | English | review | NA | NA | ALL |
| Yu et al. (2021) [102] | China | English | original research | NO | NO | US |
| Zhang et al. (2019) [103] | USA | English | review | NA | NA | ALL |
| Zhang et al. (2021) [104] | China | English | review | NA | NA | US |
| Zhang et al. (2022) [105] | China | English | review | NA | NA | ALL |
| Zhou et al. (2021) [106] | China | English | original research | UR | YES | MG |
| Zou et al. (2019) [107] | China | English | review | NA | NA | MG |

MG: Digital mammography, US: Ultrasound, MRI: Magnetic Resonance Imaging, DBT: Digital breast tomosynthesis, ALL: includes breast imaging in general, NA: not applicable, UR: under request, YES: data or code available, NO: data or code not available
*Language: Danish and German papers have been translated into English. Chinese papers were not included.

1. Akkus Z, Cai J, Boonrod A, et al (2019) A Survey of Deep-Learning Applications in Ultrasound: Artificial Intelligence–Powered Ultrasound for Improving Clinical Workflow. Journal of the American College of Radiology. DOI:10.1016/j.jacr.2019.06.004

2. Ayer T, Chen Q, Burnside ES (2013) Artificial neural networks in mammography interpretation and diagnostic decision making. Computational and Mathematical Methods in Medicine. DOI:10.1155/2013/832509

3. Bahl M (2020) Artificial intelligence: A primer for breast imaging radiologists. Journal of Breast Imaging. DOI:10.1093/jbi/wbaa033

4. Bai J, Posner R, Wang T, Yang C, Nabavi S (2021) Applying deep learning in digital breast tomosynthesis for automatic breast cancer detection: A review. Medical Image Analysis. DOI:10.1016/j.media.2021.102049

5. Balkenende L, Teuwen J, Mann RM (2022) Application of Deep Learning in Breast Cancer Imaging. Seminars in Nuclear Medicine. DOI:10.1053/j.semnuclmed.2022.02.003

6. Baltzer PAT (2021) Künstliche Intelligenz in der Mammadiagnostik. Der Radiologe. DOI:10.1007/s00117-020-00802-2

7. Batchu S, Liu F, Amireh A, Waller J, Umair M (2021) A Review of Applications of Machine Learning in Mammography and Future Challenges. Oncology. DOI:10.1159/000515698

8. Bennani-Baiti B, Baltzer PAT (2020) Künstliche Intelligenz in der Mammadiagnostik. Der Radiologe. DOI:10.1007/s00117-019-00615-y

9. Benndorf M, Burnside E, Herda C, Langer M, Kotter E (2015) External validation of a publicly available computer assisted diagnostic tool for mammographic mass lesions with two high prevalence research datasets. MEDICAL PHYSICS. DOI:10.1118/1.4927260

10. Bi WL, Hosny A, Schabath MB, et al (2019) Artificial intelligence in cancer imaging: Clinical challenges and applications. CA: A Cancer Journal for Clinicians. DOI:10.3322/caac.21552

11. Bitencourt A, Naranjo I, Lo Gullo R, Saccarelli C, Pinker K (2021) AI-enhanced breast imaging: Where are we and where are we heading? European Journal of Radiology. DOI:10.1016/j.ejrad.2021.109882

12. Calisto F, Santiago C, Nunes N, Nascimento J (2021) Introduction of human-centric AI assistant to aid radiologists for multimodal breast image classification. INTERNATIONAL JOURNAL OF HUMAN-COMPUTER STUDIES. DOI:10.1016/j.ijhcs.2021.102607

13. Chan H-P, Samala RK, Hadjiiski LM (2020) CAD and AI for breast cancer—recent development and challenges. The British Journal of Radiology. DOI:10.1259/bjr.20190580

14. Chang Y-W, An JK, Choi N, et al (2022) Artificial Intelligence for Breast Cancer Screening in Mammography (AI-STREAM): A Prospective Multicenter Study Design in Korea Using AI-Based CADe/x. Journal of Breast Cancer. DOI:10.4048/jbc.2022.25.e4

15. Chaudhary V, Chaudhari S (2021) Application of Machine Learning in Breast Cancer Diagnosis: A Review. In: 2021 IEEE Bombay Section Signature Conference (IBSSC). pp 1–6. DOI:10.1109/IBSSC53889.2021.9673373

16. Cheung HMC, Rubin D (2021) Challenges and opportunities for artificial intelligence in oncological imaging. Clinical Radiology. DOI:10.1016/j.crad.2021.03.009

17. Choy G, Khalilzadeh O, Michalski M, et al (2018) Current Applications and Future Impact of Machine Learning in Radiology. RADIOLOGY. DOI:10.1148/radiol.2018171820

18. Ciritsis A, Rossi C, Eberhard M, Marcon M, Becker AS, Boss A (2019) Automatic classification of ultrasound breast lesions using a deep convolutional neural network mimicking human decision-making. European Radiology. DOI:10.1007/s00330-019-06118-7

19. Cuocolo R, Caruso M, Perillo T, Ugga L, Petretta M (2020) Machine Learning in oncology: A clinical appraisal. CANCER LETTERS. DOI:10.1016/j.canlet.2020.03.032

20. Dai J, Lei S, Dong L, et al (2021) More Reliable AI Solution: Breast Ultrasound Diagnosis Using Multi-AI Combination. arXiv:210102639 [cs]

21. Daimiel Naranjo I, Gibbs P, Reiner JS, et al (2022) Breast Lesion Classification with Multiparametric Breast MRI Using Radiomics and Machine Learning: A Comparison with Radiologists’ Performance. Cancers. DOI:10.3390/cancers14071743

22. Davatzikos C, Sotiras A, Fan Y, et al (2019) Precision diagnostics based on machine learning-derived imaging signatures. Magnetic Resonance Imaging. DOI:10.1016/j.mri.2019.04.012

23. Deshmukh PB, Kashyap KL (2021) Research Challenges in Breast Cancer Classification through Medical Imaging Modalities using Machine Learning. In: 2021 International Conference on Industrial Electronics Research and Applications (ICIERA). IEEE, New Delhi, India, pp 1–5. DOI:10.1109/ICIERA53202.2021.9726746

24. Du R, Chen Y, Li T, Shi L, Fei Z, Li Y (2022) Discrimination of Breast Cancer Based on Ultrasound Images and Convolutional Neural Network. J Oncol 2022:7733583

25. Elbatel M (2022) Mammograms Classification: A Review. arXiv:220303618 [cs, eess]

26. Elhakim MT, Graumann O, Larsen LB, Nielsen M, Rasmussen BS (2020) Artificial intelligence for cancer detection in breast cancer screening. Ugeskr Laeger 182:

27. Fazal MI, Patel ME, Tye J, Gupta Y (2018) The past, present and future role of artificial intelligence in imaging. European Journal of Radiology. DOI:10.1016/j.ejrad.2018.06.020

28. Freeman K, Geppert J, Stinton C, et al (2021) Use of artificial intelligence for image analysis in breast cancer screening programmes: systematic review of test accuracy. BMJ (Clinical research ed). DOI:10.1136/bmj.n1872

29. Fuchsjäger M (2019) Is the future of breast imaging with AI? European Radiology. DOI:10.1007/s00330-019-06286-6

30. Fujioka T, Mori M, Kubota K, et al (2020) The Utility of Deep Learning in Breast Ultrasonic Imaging: A Review. Diagnostics 10:1055

31. Gao Y, Geras KJ, Lewin AA, Moy L (2019) New Frontiers: An Update on Computer-Aided Diagnosis for Breast Imaging in the Age of Artificial Intelligence. AJR American journal of roentgenology. DOI:10.2214/AJR.18.20392

32. Gardezi SJS, Elazab A, Lei B, Wang T (2019) Breast Cancer Detection and Diagnosis Using Mammographic Data: Systematic Review. Journal of Medical Internet Research. DOI:10.2196/14464

33. Geras KJ, Mann RM, Moy L (2019) Artificial Intelligence for Mammography and Digital Breast Tomosynthesis: Current Concepts and Future Perspectives. Radiology. DOI:10.1148/radiol.2019182627

34. Grimm L (2021) Radiomics: A Primer for Breast Radiologists. JOURNAL OF BREAST IMAGING. DOI:10.1093/jbi/wbab014

35. Halim A, Andrew A, Yasin M, et al (2021) Existing and Emerging Breast Cancer Detection Technologies and Its Challenges: A Review. Applied Sciences. DOI:10.3390/app112210753

36. Hameed BMZ, Prerepa G, Patil V, et al (2021) Engineering and clinical use of artificial intelligence (AI) with machine learning and data science advancements: radiology leading the way for future. Therapeutic Advances in Urology. DOI:10.1177/17562872211044880

37. Han S, Kang HK, Jeong JY, et al (2017) A deep learning framework for supporting the classification of breast lesions in ultrasound images. Physics in medicine and biology. DOI:10.1088/1361-6560/aa82ec

38. Harvey H, Karpati E, Khara G, et al (2019) The Role of Deep Learning in Breast Screening. Current Breast Cancer Reports. DOI:10.1007/s12609-019-0301-7

39. Heller SL, Wegener M, Babb JS, Gao Y (2021) Can an Artificial Intelligence Decision Aid Decrease False-Positive Breast Biopsies? Ultrasound Quarterly. DOI:10.1097/RUQ.0000000000000550

40. Hou R, Peng Y, Grimm LJ, et al (2021) Anomaly Detection of Calcifications in Mammography Based on 11,000 Negative Cases. IEEE Transactions on Biomedical Engineering. DOI:10.1109/TBME.2021.3126281

41. Houssami N, Kirkpatrick-Jones G, Noguchi N, Lee CI (2019) Artificial Intelligence (AI) for the early detection of breast cancer: a scoping review to assess AI’s potential in breast screening practice. Expert Review of Medical Devices. DOI:10.1080/17434440.2019.1610387

42. Hu Q, Giger ML (2021) Clinical Artificial Intelligence Applications: Breast Imaging. Radiologic Clinics of North America. DOI:10.1016/j.rcl.2021.07.010

43. Huang S, Yang J, Fong S, Zhao Q (2020) Artificial intelligence in cancer diagnosis and prognosis: Opportunities and challenges. Cancer Letters. DOI:10.1016/j.canlet.2019.12.007

44. Jiménez-Sánchez A, Tardy M, Ballester MAG, Mateus D, Piella G (2021) Memory-aware curriculum federated learning for breast cancer classification. arXiv:210702504 [cs]

45. Johansson G, Olsson C, Smith F, Edegran M, Björk-Eriksson T (2021) AI-aided detection of malignant lesions in mammography screening - evaluation of a program in clinical practice. BJR Open 3:20200063

46. Kim S, Lee H, Kim H, Ro Y (2018) ICADx: Interpretable computer aided diagnosis of breast masses. In: Medical Imaging 2018: Computer-Aided Diagnosis. p 73. DOI:10.1117/12.2293570

47. Kim Y, Kang BJ, Lee JM, Kim SH (2019) Comparison of the Diagnostic Performance of Breast Ultrasound and CAD Using BI-RADS Descriptors and Quantitative Variables. Iranian Journal of Radiology. DOI:10.5812/iranjradiol.67729

48. Kim J, Kim HJ, Kim C, Kim WH (2021) Artificial intelligence in breast ultrasonography. Ultrasonography. DOI:10.14366/usg.20117

49. Komatsu M, Sakai A, Dozen A, et al (2021) Towards Clinical Application of Artificial Intelligence in Ultrasound Imaging. Biomedicines. DOI:10.3390/biomedicines9070720

50. Kyono T, Gilbert FJ, van der Schaar M (2018) MAMMO: A Deep Learning Solution for Facilitating Radiologist-Machine Collaboration in Breast Cancer Diagnosis. arXiv:181102661 [cs, stat]

51. Lamb LR, Lehman CD, Gastounioti A, Conant EF, Bahl M (2022) Artificial Intelligence (AI) for Screening Mammography, From the AJR Special Series on AI Applications. American Journal of Roentgenology. DOI:10.2214/AJR.21.27071

52. Lassau N, Estienne T, de Vomecourt P, et al (2019) Five simultaneous artificial intelligence data challenges on ultrasound, CT, and MRI. Diagnostic and interventional imaging. DOI:10.1016/j.diii.2019.02.001

53. Lassau N, Bousaid I, Chouzenoux E, et al (2021) Three artificial intelligence data challenges based on CT and ultrasound. Diagnostic and interventional imaging. DOI:10.1016/j.diii.2021.06.005

54. Le EPV, Wang Y, Huang Y, Hickman S, Gilbert FJ (2019) Artificial intelligence in breast imaging. Clinical Radiology. DOI:10.1016/j.crad.2019.02.006

55. Lee CI, Houssami N, Elmore JG, Buist DSM (2020) Pathways to breast cancer screening artificial intelligence algorithm validation. Breast. DOI:10.1016/j.breast.2019.09.005

56. Lee SH, Park H, Ko ES (2020) Radiomics in Breast Imaging from Techniques to Clinical Applications: A Review. Korean journal of radiology. DOI:10.3348/kjr.2019.0855

57. Lei Y-M, Yin M, Yu M-H, et al (2021) Artificial Intelligence in Medical Imaging of the Breast. Frontiers in Oncology. DOI:10.3389/fonc.2021.600557

58. Li H, Ye J, Liu H, et al (2021) Application of deep learning in the detection of breast lesions with four different breast densities. Cancer Medicine. DOI:10.1002/cam4.4042

59. Lyu SY, Zhang Y, Zhang MW, et al (2022) Diagnostic value of artificial intelligence automatic detection systems for breast BI-RADS 4 nodules. World journal of clinical cases. DOI:10.12998/wjcc.v10.i2.518

60. Makino T, Jastrzebski S, Oleszkiewicz W, et al (2020) Differences between human and machine perception in medical diagnosis. arXiv:201114036 [cs, eess]

61. Mann RM, Hooley R, Barr RG, Moy L (2020) Novel Approaches to Screening for Breast Cancer. Radiology. DOI:10.1148/radiol.2020200172

62. Maqsood S, Damasevicius R, Maskeliunas R (2022) TTCNN: A Breast Cancer Detection and Classification towards Computer-Aided Diagnosis Using Digital Mammography in Early Stages. Applied Sciences. DOI:10.3390/app12073273

63. Massat MB (2019) The integration of artificially intelligent technologies with breast imaging. Applied Radiology. DOI:10.37549/AR2602

64. Masud R, Al-Rei M, Lokker C (2019) Computer-Aided Detection for Breast Cancer Screening in Clinical Settings: Scoping Review. JMIR Medical Informatics. DOI:10.2196/12660

65. Meyer-Bäse A, Morra L, Meyer-Bäse U, Pinker K (2020) Current Status and Future Perspectives of Artificial Intelligence in Magnetic Resonance Breast Imaging. Contrast Media & Molecular Imaging. DOI:10.1155/2020/6805710

66. Morgan MB, Mates JL (2021) Applications of Artificial Intelligence in Breast Imaging. Radiologic Clinics of North America. DOI:10.1016/j.rcl.2020.08.007

67. European Society of Radiology (2019) What the radiologist should know about artificial intelligence - an ESR white paper. Insights into imaging. DOI:10.1186/s13244-019-0738-2

68. Nishikawa RM, Gur D (2014) CADe for Early Detection of Breast Cancer—Current Status and Why We Need to Continue to Explore New Approaches. Academic Radiology. DOI:10.1016/j.acra.2014.05.018

69. Ou WC, Polat D, Dogan BE (2021) Deep learning in breast radiology: current progress and future directions. European radiology. DOI:10.1007/s00330-020-07640-9

70. Panayides A, Amini A, Filipovic N, et al (2020) AI in Medical Imaging Informatics: Current Challenges and Future Directions. IEEE JOURNAL OF BIOMEDICAL AND HEALTH INFORMATICS. DOI:10.1109/JBHI.2020.2991043

71. Pang T, Wong J, Ng W, Chan C (2020) Deep learning radiomics in breast cancer with different modalities: Overview and future. Expert Systems with Applications. DOI:10.1016/j.eswa.2020.113501

72. Pedemonte S, Tsue T, Mombourquette B, et al (2022) A deep learning algorithm for reducing false positives in screening mammography. arXiv:220406671 [cs]

73. Pesapane F, Rotili A, Agazzi GM, et al (2021) Recent Radiomics Advancements in Breast Cancer: Lessons and Pitfalls for the Next Future. Current Oncology. DOI:10.3390/curroncol28040217

74. Qian X, Pei J, Zheng H, et al (2021) Prospective assessment of breast cancer risk from multimodal multiview ultrasound images via clinically applicable deep learning. Nature biomedical engineering. DOI:10.1038/s41551-021-00711-2

75. Qiao M, Liu C, Li Z, et al (2022) Breast Tumor Classification Based on MRI-US Images by Disentangling Modality Features. IEEE Journal of Biomedical and Health Informatics. DOI:10.1109/JBHI.2022.3140236

76. Reardon S (2019) Rise of Robot Radiologists. Nature. DOI:10.1038/d41586-019-03847-z

77. Rizzi M, D’aloia M (2014) COMPUTER AIDED SYSTEM FOR BREAST CANCER DIAGNOSIS. Biomedical Engineering - Applications, Basis and Communications. DOI:10.4015/S1016237214500331

78. Rodriguez-Ruiz A, Lång K, Gubern-Merida A, et al (2019) Can we reduce the workload of mammographic screening by automatic identification of normal exams with artificial intelligence? A feasibility study. European Radiology. DOI:10.1007/s00330-019-06186-9

79. Rodriguez-Ruiz A, Lång K, Gubern-Merida A, et al (2019) Stand-Alone Artificial Intelligence for Breast Cancer Detection in Mammography: Comparison With 101 Radiologists. JNCI: Journal of the National Cancer Institute. DOI:10.1093/jnci/djy222

80. Sahiner B, Pezeshk A, Hadjiiski L, et al (2019) Deep learning in medical imaging and radiation therapy. MEDICAL PHYSICS. DOI:10.1002/mp.13264

81. Samala RK, Chan HP, Hadjiiski L, Helvie MA (2021) Risks of feature leakage and sample size dependencies in deep feature extraction for breast mass classification. Medical physics. DOI:10.1002/mp.14678

82. Satake H, Ishigaki S, Ito R, Naganawa S (2022) Radiomics in breast MRI: current progress toward clinical application in the era of artificial intelligence. La Radiologia Medica. DOI:10.1007/s11547-021-01423-y

83. Sato M, Kawai M, Nishino Y, Shibuya D, Ohuchi N, Ishibashi T (2014) Cost-effectiveness analysis for breast cancer screening: double reading versus single + CAD reading. Breast Cancer. DOI:10.1007/s12282-012-0423-5

84. Schaffter T, Buist DSM, Lee CI, et al (2020) Evaluation of Combined Artificial Intelligence and Radiologist Assessment to Interpret Screening Mammograms. JAMA Network Open. DOI:10.1001/jamanetworkopen.2020.0265

85. Sechopoulos I, Teuwen J, Mann R (2021) Artificial intelligence for breast cancer detection in mammography and digital breast tomosynthesis: State of the art. Seminars in Cancer Biology. DOI:10.1016/j.semcancer.2020.06.002

86. Shan C, Tan T, Han J, Huang D (2021) Ultrasound tissue classification: a review. ARTIFICIAL INTELLIGENCE REVIEW. DOI:10.1007/s10462-020-09920-8

87. Shastry KA, Sanjay HA (2022) Cancer diagnosis using artificial intelligence: a review. Artificial Intelligence Review. DOI:10.1007/s10462-021-10074-4

88. Shen Y-T, Chen L, Yue W-W, Xu H-X (2021) Artificial intelligence in ultrasound. European Journal of Radiology. DOI:10.1016/j.ejrad.2021.109717

89. Shoshan Y, Bakalo R, Gilboa-Solomon F, et al (2022) Artificial Intelligence for Reducing Workload in Breast Cancer Screening with Digital Breast Tomosynthesis. Radiology. DOI:10.1148/radiol.211105

90. Song L, Hsu W, Xu J, van der Schaar M (2016) Using Contextual Learning to Improve Diagnostic Accuracy: Application in Breast Cancer Screening. IEEE JOURNAL OF BIOMEDICAL AND HEALTH INFORMATICS. DOI:10.1109/JBHI.2015.2414934

91. Subuhana N, Aysha Rega S, Sundar S (2021) Deep Learning Techniques for Breast Cancer Analysis: A Review. In: 2021 Fourth International Conference on Microelectronics, Signals & Systems (ICMSS). IEEE, Kollam, India, pp 1–6. DOI:10.1109/ICMSS53060.2021.9673651

92. Tadavarthi Y, Vey B, Krupinski E, et al (2020) The State of Radiology AI: Considerations for Purchase Decisions and Current Market Offerings. Radiology: Artificial intelligence. DOI:10.1148/ryai.2020200004

93. Tagliafico AS, Piana M, Schenone D, Lai R, Massone AM, Houssami N (2019) Overview of radiomics in breast cancer diagnosis and prognostication. The Breast : official journal of the European Society of Mastology. DOI:10.1016/j.breast.2019.10.018

94. Tartar M, Le L, Watanabe AT, Enomoto AJ (2021) Artificial Intelligence Support for Mammography: In-Practice Clinical Experience. Journal of the American College of Radiology : JACR. DOI:10.1016/j.jacr.2021.09.016

95. Tasdemir S, Tasdemir K, Aydin Z (2020) A review of mammographic region of interest classification. WIREs Data Mining and Knowledge Discovery. DOI:10.1002/widm.1357

96. Thomassin-Naggara I, Balleyguier C, Ceugnart L, et al (2019) Artificial intelligence and breast screening: French Radiology Community position paper. Diagnostic and Interventional Imaging. DOI:10.1016/j.diii.2019.08.005

97. Trivizakis E, Papadakis GZ, Souglakos I, et al (2020) Artificial intelligence radiogenomics for advancing precision and effectiveness in oncologic care (Review). International Journal of Oncology. DOI:10.3892/ijo.2020.5063

98. Vobugari N, Raja V, Sethi U, Gandhi K, Raja K, Surani SR (2022) Advancements in Oncology with Artificial Intelligence—A Review Article. Cancers. DOI:10.3390/cancers14051349

99. Wang X, Liang G, Zhang Y, Blanton H, Bessinger Z, Jacobs N (2020) Inconsistent Performance of Deep Learning Models on Mammogram Classification. Journal of the American College of Radiology : JACR. DOI:10.1016/j.jacr.2020.01.006

100. Wang Z, Luo Y, Xin J, et al (2020) Computer-Aided Diagnosis Based on Extreme Learning Machine: A Review. IEEE ACCESS. DOI:10.1109/ACCESS.2020.3012093

101. Wichmann JL, Willemink MJ, De Cecco CN (2020) Artificial Intelligence and Machine Learning in Radiology: Current State and Considerations for Routine Clinical Implementation. Investigative Radiology. DOI:10.1097/RLI.0000000000000673

102. Yu T-F, He W, Gan C-G, et al (2021) Deep learning applied to two-dimensional color Doppler flow imaging ultrasound images significantly improves diagnostic performance in the classification of breast masses: a multicenter study. Chinese Medical Journal. DOI:10.1097/CM9.0000000000001329

103. Zhang Z, Sejdić E (2019) Radiological images and machine learning: Trends, perspectives, and prospects. Computers in Biology and Medicine. DOI:10.1016/j.compbiomed.2019.02.017

104. Zhang D, Jiang F, Yin R, et al (2021) A Review of the Role of the S-Detect Computer-Aided Diagnostic Ultrasound System in the Evaluation of Benign and Malignant Breast and Thyroid Masses. Medical Science Monitor: International Medical Journal of Experimental and Clinical Research. DOI:10.12659/MSM.931957

105. Zhang X, Zhang Y, Zhang G, et al (2022) Deep Learning With Radiomics for Disease Diagnosis and Treatment: Challenges and Potential. Frontiers in Oncology. DOI:10.3389/fonc.2022.773840

106. Zhou Q, Zuley M, Guo Y, et al (2021) A machine and human reader study on AI diagnosis model safety under attacks of adversarial images. Nature communications. DOI:10.1038/s41467-021-27577-x

107. Zou L, Yu S, Meng T, Zhang Z, Liang X, Xie Y (2019) A Technical Review of Convolutional Neural Network-Based Mammographic Breast Cancer Diagnosis. Computational and Mathematical Methods in Medicine. DOI:10.1155/2019/6509357
